# RNA-based thermoregulation of a *Campylobacter jejuni* zinc resistance determinant

**DOI:** 10.1371/journal.ppat.1009008

**Published:** 2020-10-16

**Authors:** Heba Barnawi, Nader Masri, Natasha Hussain, Bushra Al-Lawati, Evita Mayasari, Aleksandra Gulbicka, Adrian J. Jervis, Min-Hsuan Huang, Jennifer S. Cavet, Dennis Linton

**Affiliations:** 1 The Lydia Becker Institute of Immunology and Inflammation, Faculty of Biology, Medicine, and Health, University of Manchester, Manchester Academic Health Science Centre, Manchester, United Kingdom; 2 Microbiology Department, Faculty of Medicine, Universitas Sumatera Utara, Indonesia; 3 Manchester Centre for Synthetic Biology of Fine and Speciality Chemicals (SYNBIOCHEM), Manchester Institute of Biotechnology, The University of Manchester, Manchester, United Kingdom; University of Texas Southwestern Medical Center, UNITED STATES

## Abstract

RNA thermometers (RNATs) trigger bacterial virulence factor expression in response to the temperature shift on entering a warm-blooded host. At lower temperatures these secondary structures sequester ribosome-binding sites (RBSs) to prevent translation initiation, whereas at elevated temperatures they “melt” allowing translation. *Campylobacter jejuni* is the leading bacterial cause of human gastroenteritis worldwide yet little is known about how it interacts with the host including host induced gene regulation. Here we demonstrate that an RNAT regulates a *C*. *jejuni* gene, Cj1163c or *czcD*, encoding a member of the Cation Diffusion Facilitator family. The *czcD* upstream untranslated region contains a predicted stem loop within the mRNA that sequesters the RBS to inhibit translation at temperatures below 37°C. Mutations that disrupt or enhance predicted secondary structure have significant and predictable effects on temperature regulation. We also show that in an RNAT independent manner, CzcD expression is induced by Zn(II). Mutants lacking *czcD* are hypersensitive to Zn(II) and also over-accumulate Zn(II) relative to wild-type, all consistent with CzcD functioning as a Zn(II) exporter. Importantly, we demonstrate that *C*. *jejuni* Zn(II)-tolerance at 32°C, a temperature at which the RNAT limits CzcD production, is increased by RNAT disruption. Finally we show that *czcD* inactivation attenuates larval killing in a Galleria infection model and that at 32°C disrupting RNAT secondary structure to allow CzcD production can enhance killing. We hypothesise that CzcD regulation by metals and temperature provides a mechanism for *C*. *jejuni* to overcome innate immune system-mediated Zn(II) toxicity in warm-blooded animal hosts.

## Author summary

Sensing the elevated warm-blooded host body temperature through structural changes in so-called RNA thermometers is a recognised virulence strategy in bacterial pathogens and allows appropriate production of virulence factors in the host environment. We demonstrated that this regulatory mechanism is used by *Campylobacter jejuni*, a globally significant cause of human gastroenteritis, to control production of the CzcD protein that we show acts as a Zn(II) exporter providing tolerance to high levels of Zn(II). From published data, Zn(II) exporters are known to enable bacterial pathogens to overcome host-induced Zn(II) poisoning—a newly discovered innate immune response to infection. Indeed using a simple larval infection model we demonstrated that CzcD enhances larval killing and disrupting the RNA thermometer to allow CzcD production also enhanced killing at lower temperatures. These findings indicate that CzcD temperature regulation enables *C*. *jejuni* to overcome host induced Zn(II) toxicity during colonisation of animal hosts including humans. Our results open up a novel area of the host-pathogen interface for investigation in this poorly understood human pathogen. It may also be possible to exploit these findings to develop metal-based intervention strategies to reduce both levels of *Campylobacter* in the food chain and consequent zoonotic infections.

## Introduction

The Gram-negative bacterium *Campylobacter jejuni* is a globally significant cause of gastroenteritis with an estimated annual incidence of 166 million cases [1]. In developing countries disease is often endemic with symptomatic infections in infants but not in older age groups, presumably due to development of acquired immunity through repeated exposure [2]. In developed countries *C*. *jejuni* infections are typically symptomatic, occur sporadically across all age groups and are commonly transmitted via food derived from birds and animals where it is considered to be a commensal. The consumption of poultry, in particular chicken, is a major source of infection [3]. Disease symptoms typically comprise a bout of self-limiting inflammatory diarrhoea and stomach cramps although in the young, elderly and immunosuppressed the symptoms can be more severe and even life threatening. There are also rare but serious infection sequelae including the autoimmune mediated peripheral neuropathy Guillain-Barré syndrome [4]. At the cellular level the ability to adhere to and invade gut epithelial cells is an established important first step in human infections and is associated with inflammation and phagocyte infiltration [5–7]. The public health significance of campylobacteriosis underlines the urgent need for new strategies to reduce infections. However, the mechanisms employed by *C*. *jejuni* to adapt to its intra- and extracellular lifestyles and to colonise and infect its hosts are relatively poorly understood.

Zn(II) is an essential co-factor for many enzymes, however, elevated levels of Zn(II) can also be toxic and hence precise intracellular Zn(II) homeostasis is required for bacterial survival [8]. To inhibit bacterial proliferation, host innate immune defences exploit this need and both restrict Zn(II)-availability, in a process termed “nutritional immunity”, and poison bacteria with antimicrobial concentrations of Zn(II) [9–11]. For example, the proinflammatory cytokine IL-6 causes increased abundance of the Zn(II) transporter Zip14 at the plasma membrane of hepatocytes, resulting in increased hepatic Zn(II) accumulation which likely contributes to the reduced serum Zn(II) levels that occur during the acute phase response to infection [12]. The inflammatory S100 protein calprotectin also contributes to bacterial killing at certain sites of infection by sequestering metals [including Zn(II) and Mn(II)] following its copious release from neutrophils [13]. Nonetheless many bacterial pathogens can overcome this host mediated metal-limitation via Zn(II) uptake systems (for example [14–16]), and notably *C*. *jejuni* possesses an ATP-binding cassette (ABC) Zn(II)-importer needed for intestinal survival [17]. Direct Zn(II) toxicity has also been documented as a host antimicrobial strategy against several pathogens. For example *Mycobacterium tuberculosis* and *Salmonella enterica* serovar Typhimurium are subjected to Zn(II) intoxication in macrophage phagosomes, where Zn(II) toxicity can act synergistically with the antimicrobial effects of copper which is also targeted to this compartment [18,19]. Zn(II) levels are also reported to increase in the various host niches colonised by *Streptococcus pneumoniae* to inhibit bacterial growth [20] and dietary Zn(II) restriction impacts on the ability of phagocytes to control *S*. *pneumonia* infection by Zn(II) intoxication [21]. Furthermore, high levels of both calprotectin and Zn(II) are found at skin sites colonised by group A *Streptococcus*, with bacterial control being associated with both extracellular Zn(II) chelation by calprotectin and intracellular Zn(II) intoxication following phagocytosis [22]. Consistent with host mediated Zn(II)-toxicity contributing to immune defences, the disruption of genes encoding Zn(II)-exporting P_1B_-type ATPases (often designated ZntA) or Cation Diffusion Facilitators (CDFs)/CzcD proteins in these pathogens reduces virulence [11]. To date, nothing is known about the mechanisms that might protect *C*. *jejuni* from Zn(II)-toxicity during infection. Here we report that Cj1163c (herein designated CzcD) recently shown to enhance colonisation in a mouse intestinal infection model [23] is a CDF metal-effluxer with a primary role in Zn(II)-resistance. Moreover, we reveal that *C*. *jejuni* CzcD is under the control of an RNA thermal switch that restricts its production to the elevated temperatures of its warm-blooded hosts.

RNA thermometers (RNATs) are temperature-sensing elements located within 5’ untranslated regions of mRNAs [24]. They typically respond to temperature through sequestering ribosome binding sites within the secondary structure at low temperatures and as the temperature increases the structure “melts” allowing ribosome access and consequent translation. RNATs are increasingly associated with bacterial virulence and are thought to enable rapid production of key virulence proteins on sensing elevated host body temperatures (reviewed in [25]). Examples include virulence associated transcriptional regulators from *Listeria monocytogenes* [26], *Vibrio cholerae* [27], *Pseudomonas aeruginosa* [28] and *Yersinia pseudotuberculosis* [29]; genes involved in capsule biosynthesis and production of Factor H binding protein in *Neisseria meningitidis* [30,31]; a complement protein-binding lipoprotein from *Leptospira interrogans* [32] and a cytotoxin from *Y*. *pseudotuberculosis* [33]. The only examples, to date, of bacterial RNAT-regulated genes associated with metal handling are *Shigella dysenteriae shuA* and *shuT* involved in heme uptake [34,35].

In this study, as well as functionally characterising CzcD, we have identified and characterised an RNAT in the intergenic untranslated region immediately upstream of *czcD* that is responsible for increased CzcD expression at elevated host body temperatures (42°C in chickens and 37°C in humans) to confer temperature-dependent *C*. *jejuni* Zn(II)-resistance. To our knowledge, this is the first RNAT to be described in *C*. *jejuni* and the first to be associated with Zn(II) resistance. Finally, we demonstrate that *czcD* inactivation attenuates killing in a simple *Galleria mellonella* larvae infection model and that at lower temperatures larval killing is enhanced when the *czcD* RNAT is mutated to allow increased CzcD production. Our findings are consistent with Zn(II)-export being important for *C*. *jejuni* host colonisation and infection by protecting against host mediated Zn(II)-toxicity.

## Results

### The 5’ untranslated region upstream of *czcD* confers temperature sensitive regulation in a heterologous host

**Fig 1:**
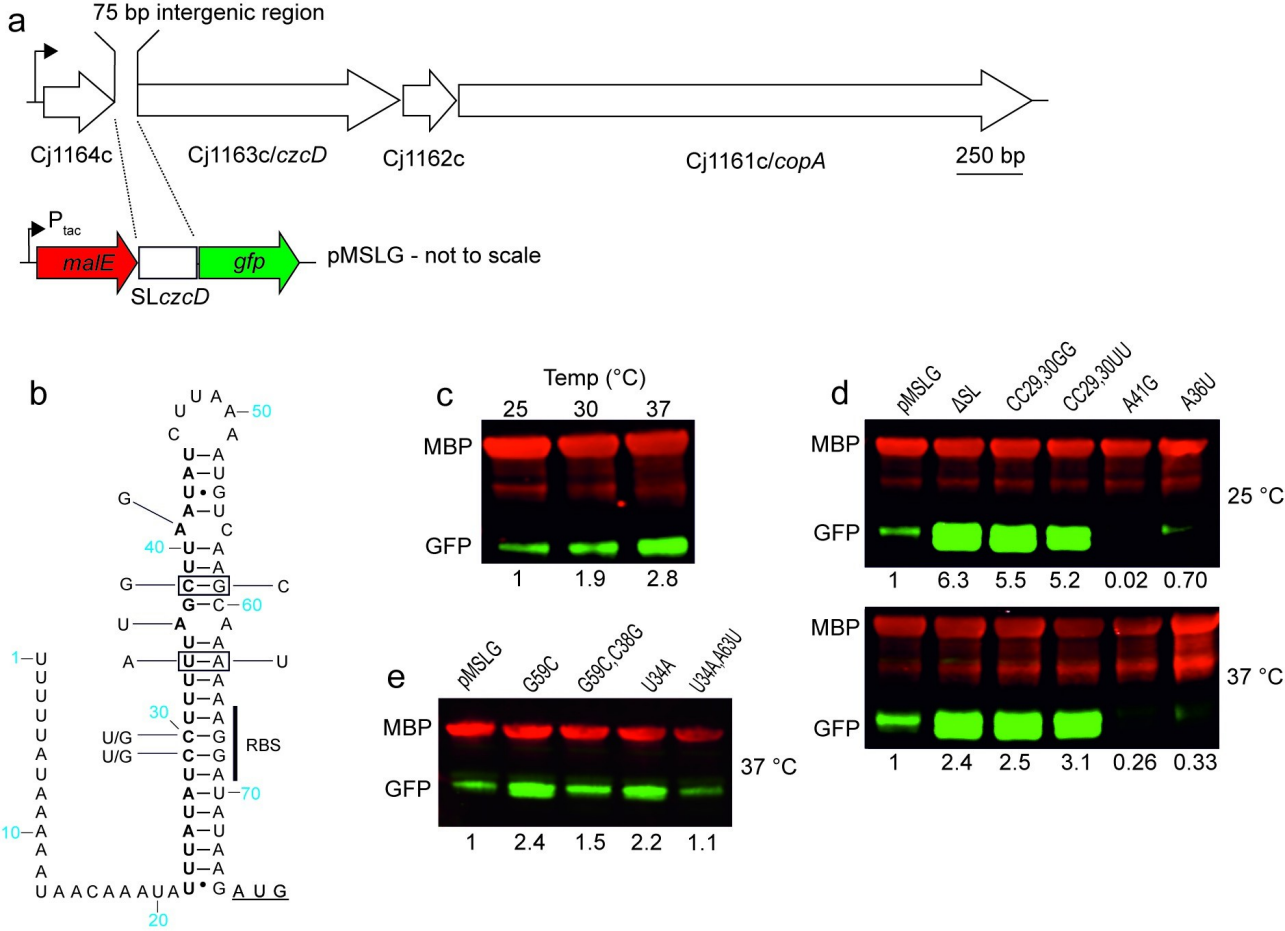
The Cj1164c/Cj1163c intergenic region mediates temperature dependent regulation of a downstream gene in *E*. *coli*. a. Diagrammatic representation of the *C*. *jejuni* NCTC 11168 operon containing the Cj1163c/*czcD* gene with the shaded arrowhead indicating the associated σ^70^ promoter identified by RNAseq analyses [36,37]. Located between Cj1164c and *czcD* is a 75 bp untranslated intergenic region (SL*czcD*) that was cloned between reporter genes *malE* (red) encoding maltose binding protein and *gfp* (green) on plasmid pMSLG downstream of the inducible *tac* promoter P_*tac*_. b. Predicted SL*czcD* secondary structure from Mfold analysis with the *czcD* start codon underlined and ribosome binding site (RBS) indicated. Site directed mutations predicted to enhance (A41G and A36U) or disrupt (CC29,30GG and CC29,30UU) secondary structure are indicated, whilst bases in bold indicate the region removed in the pMSLG^*ΔSL*^ construct. Boxed base pairs indicate where disrupting and subsequent compensatory mutations were introduced as indicated. c. *E*. *coli* pMSLG-containing cells were grown at 25, 30 or 37°C and standardised whole cell lysates western blotted and probed with anti-MBP (red) and anti-GFP (green) antisera. All GFP signals were standardised to the corresponding internal loading control MBP signal and normalised to the 25°C value. Relative GFP quantifications are provided below the blots. These data are representative of at least three replicate experiments. d. Similar analyses as (c) with extracts from strains containing variants of pMSLG. The pMSLG^*ΔSL*^ plasmid had the bases in bold in (b) removed. The CC29,30GG; CC29,30UU; A41G and A36U derivatives are as indicated in (b). All GFP signals were standardised to the corresponding internal loading control MBP signal and normalised to the pMSLG value. Relative GFP quantifications are provided below the blots. These data are representative of at least three replicate experiments. e. Similar analyses as (c) with compensatory mutants (boxed in b) in the predicted stem region of SL*czcD* on pMSLG. The SL*czcD* G59:C38 predicted base pair was disrupted by introducing a G59C mutation and base pairing restored with a C38G mutation. In a similar manner the U34:A63 predicted base pair was disrupted with a U34A mutation and restored with an A63U mutation. All GFP signals were standardised to the corresponding internal loading control MBP signal and normalised to the pMSLG value. Relative GFP quantifications are provided below the blots. These data are representative of at least three replicate experiments.

To identify *C*. *jejuni* genes involved in metal homeostasis we used blastp to search the NCTC 11168 predicted proteome with well characterised proteins from a number of bacterial species. Following sequence alignment and more detailed scrutiny of potential metal-binding ligands, an uncharacterised potential metal exporter Cj1163c was identified located within a four-gene operon consisting of Cj1164c through to Cj1161c (Fig 1A) with an associated upstream σ^70^-dependent promoter [36,37]. Cj1164c encodes a 10 kDa Zn(II)-finger-containing protein of unknown function, Cj1162c encodes a CopZ/Atx1 Cu(I)-chaperone like protein and Cj1161c encodes a P_1B_-type ATPase CopA that confers copper resistance [38]. Cj1163c encodes a CDF family member containing Pfam motif PF01545 characteristic of integral membrane proteins that enhance tolerance to divalent cations through functioning as efflux pumps and was previously annotated as a cation efflux protein [39]. It possesses features typical of the Zn(II) exporting CDFs [40] and as a putative Cd(II)-Zn(II)-Co(II) transporter protein we have termed it CzcD. Alignment of *C*. *jejuni* CzcD and YiiP, a structurally characterised CDF family member from *Escherichia coli* [41], demonstrated conservation of the four critical aspartate/histidine residues that form the YiiP membrane embedded Zn(II) binding active site (S1 Fig).

Notably, between Cj1164c and *czcD* is a 75 bp intergenic region containing an annotated 52 bp stem loop (nucleotides 1095030 to 1095081 of the complementary strand—GenBank accession AL111168.1) that we have termed SL*czcD*. Mfold secondary structure prediction [42] of the transcript from this intergenic region indicated that the putative *czcD* Shine-Dalgarno (SD) sequence AGGA was sequestered by base-pairing with a UCCU anti-SD sequence in SL*czcD* (Fig 1B), SD sequestration being a characteristic feature of RNATs involved in temperature-dependent post-transcriptional gene regulation. Searches of the RefSeq genome database indicated that the SL*czcD* was present in all *C*. *jejuni* but not the vast majority of *C*. *coli* strains. A sequence alignment of the SL*czcD*-containing intergenic region from 1626 *C*. *jejuni* strains identified limited nucleotide sequence variation (S2A Fig) with only minor effects on predicted secondary structure (S2B Fig) and so we consider this a conserved feature of *C*. *jejuni*.

To test whether this region conferred temperature responsiveness in the heterologous host *E*. *coli*, we created plasmid pMSLG in which the SL*czcD* region including the *czcD* start codon (reverse complement of nucleotides 1095025 to 1095084) was cloned between reporter genes *malE* and *gfp*, with the latter placed such that it used the *czcD* start codon (Fig 1A). In the resultant strain, EcpMSLG, while MBP levels did not vary with temperature, GFP levels increased as the temperature was raised from 25 to 37°C (Fig 1C) suggesting a thermoregulatory role for SL*czcD* on the downstream *gfp* gene. Deletion of residues 1 to 24 of the SL*czcD* (highlighted in bold in Fig 1B), to create the pMSLG^*ΔSL*^ construct lacking the stem loop, resulted in similar and significantly elevated levels of GFP at both 25°C and 37°C (Fig 1D). We further examined GFP production in site-directed mutants predicted to enhance or disrupt SL*czcD* secondary structure and thereby have repressing or de-repressing activity respectively (Fig 1D and 1E). The minimum free energy values of the Mfold predicted secondary structures of these variants are consistent with these predictions (S1 Table). The predicted pairing of the anti-SD:SD sequence was disrupted by substituting the two anti-SD C residues with G (to create pMSLG^*CC29*,*30GG*^), or U residues (to create pMSLG^*CC29*,*30UU*^) predicted to form weaker non-canonical base pairing. The resultant strains, EcCC29,30GG and EcCC29,30UU, produced significantly increased GFP levels relative to EcpMSLG at both 25 and 37°C (Fig 1E) highlighting the critical role of these residues in regulating production of downstream gene products. Furthermore, two repressing mutations (A36U and A41G, Fig 1B), predicted to increase SL*czcD* secondary structure through replacing small internal loops with base-pairing, resulted in decreased GFP levels in strains EcA36U and EcA41G compared to EcpMSLG (Fig 1D) demonstrating the importance of these unpaired regions in RNAT functioning. Finally, we constructed two sets of SL*czcD* mutants (G59C and U34A) that disrupted base-paired stem regions and then restored the predicted base pairing by introducing compensatory mutations (C38G and A63U respectively; Fig 1B). As predicted GFP levels increased in the EcG59C and EcU34A strains relative to EcpMSLG and were reduced to levels similar to those for the wild-type SL*czcD* following the introduction of compensatory mutations in strains EcG59C,C38G and EcU34A,A63U (Fig 1E).

To further confirm temperature responsiveness in *E*. *coli* we utilised a *lacZ* reporter gene placed immediately downstream of the SL*czcD* on a pMLBAD derivative plasmid termed pSL*lacZ* (see Materials and Methods). Derivatives of this plasmid with repressing (pSL*lacZ*A41G) and derepressing (pSL*lacZ*CC29,30GG) mutations were also constructed in a similar manner to as described above. Reporter activity measurements of cultures containing these plasmids incubated at 30, 37 and 42°C confirmed temperature responsiveness of SL*czcD*, and the effects of repressing and derepressing mutants were consistent with SL*czcD* structural prediction and western blotting data presented above (S3 Fig).

Overall these data demonstrate that the SL*czcD* acts independently of other *C*. *jejuni* factors to confer temperature responsive regulation of downstream genes in a heterologous host. Mutations predicted to disrupt or enhance secondary structure of the SL*czcD* led to predicted changes in the level of downstream gene product consistent with this region functioning as an RNAT. The ability to act independently of additional endogenous factors in a heterologous host is a notable feature of RNATs due to the conserved nature of core translational machinery that they rely on for function.

### SL*czcD*-dependent temperature regulation of CzcD production in *C*. *jejuni*

**Fig 2:**
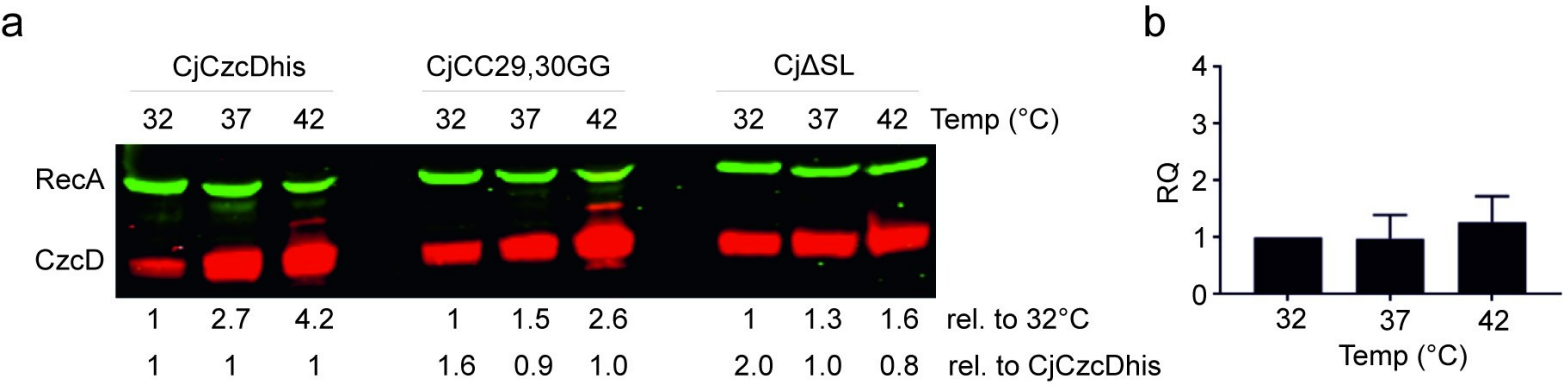
The SL*czcD* mediates temperature-dependent post transcriptional regulation of CzcD in *C*. *jejuni*. a. Western blot analysis of the *C*. *jejuni* NCTC11168 CjCzcDhis strain and derivatives with deletion of the SL*czcD* (CjΔSL) and the derepressing mutation CC29,30GG (CjCC29,30GG). Standardised whole cell lysates were probed with anti-RecA (green) as a loading control and anti-histidine (red) to detect CzcD. Cultures were grown for 24 hours in MH broth at the temperatures indicated. All CzcD signals were standardised to the corresponding internal RecA loading control signal and normalised. Relative CzcD quantifications are provided below the blots normalised to the corresponding 32°C CzcD value for each of the three strains to allow effect of temperature to be quantified (upper row) or to the CjCzcDhis value at each temperature allowing comparison of CjΔSL and CjCC29,30GG strains with CjCzcDhis (lower row). These data are representative of at least three replicate experiments. b. RT-qPCR analysis of *czcD* expression levels for strain CjCzcDhis grown for 24 hours in MH broth at the temperatures indicated. The CT values were normalised against the data at 32°C. Data are the mean of three biological replicates with standard error indicated. Comparison of ΔΔCt mean values by t test gave non-significant p values of 0.90 (37 vs 32°C) and 0.78 (42 vs 32°C).

To investigate CzcD temperature regulation in *C*. *jejuni* directly we produced an NCTC 11168 strain that produces hexa-histidine tagged CzcD (CjCzcDhis) from the native locus (see Materials and Methods) and tested for CzcD thermoregulation. As predicted, CzcDhis levels increased with temperature from 32 to 37°C, and again from 37 to 42°C (Fig 2A). Importantly, this was not due to a significant increase in transcript abundance as measured by RT-qPCR (Fig 2B), consistent with RNAT-mediated regulation at the translational level. To determine if the SL*czcD* was responsible for CzcD temperature regulation, we created a derivative of CjCzcDhis in which the SL*czcD* was removed (CjΔSL). In this strain CzcD temperature regulation was reduced (Fig 2A). A strain with de-repressing mutations CC29,30GG in the SL*czcD*, termed CjCC29,30GG, retained only a limited degree of temperature induction (Fig 2A). These data demonstrate the role of the *cj1164*-*czcD* intergenic region in temperature regulation of CzcD production and are consistent with the predicted stem loop region, SL*czcD*, within this intergenic region acting as an RNAT.

### CzcD is a Zn(II) resistance determinant

**Fig 3:**
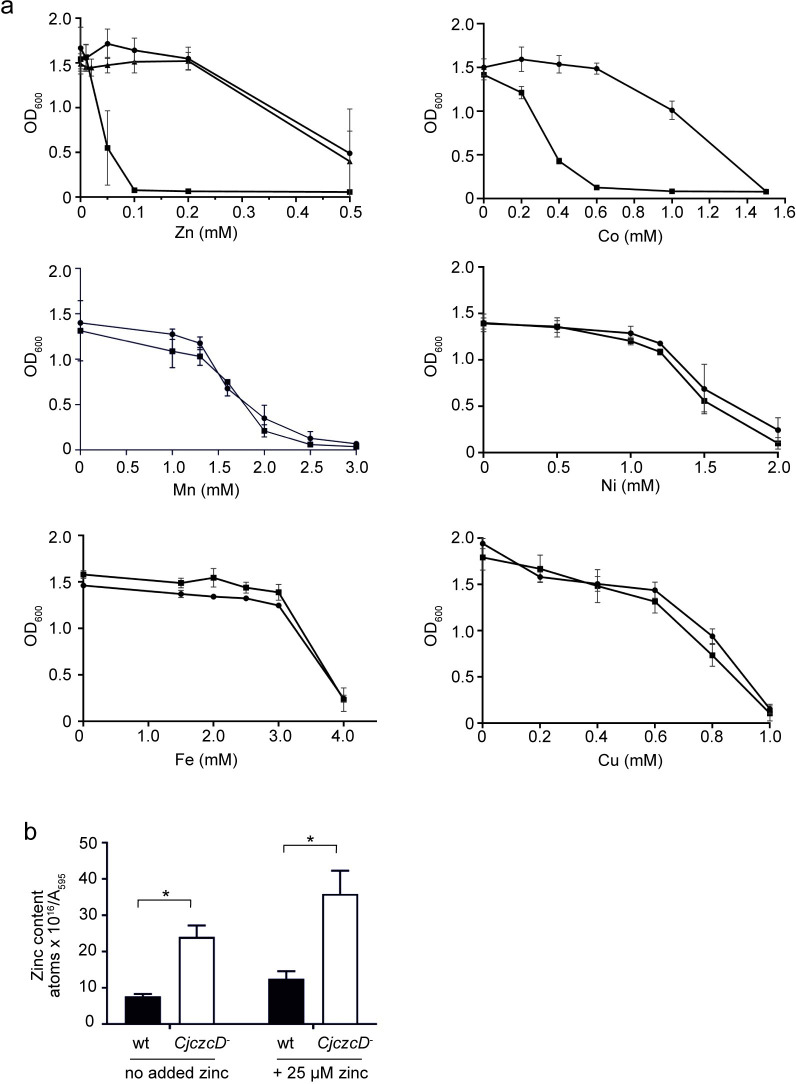
CzcD is a Zn(II) exporter and Zn(II)/Co(II) resistance determinant. a. *C*. *jejuni* NCTC 11168 (circles), Cj*czcD*^*-*^ (squares) and the genetically complemented Cj*czcD*^*+*^ strain (triangles) were grown for 24 hours at 37°C in MH broth from a starting OD_600_ of 0.05 with varying concentrations of metals and the OD_600_ measured. For the zinc experiment, data for all three strains are presented whilst for other metals only the wild type and Cj*czcD*^-^ strain are included. Data points are the mean of three biological repeats, each with three technical repeats and error bars represent standard error of the biological repeats. b. Cellular Zn(II) content of *C*. *jejuni* NCTC 11168 (wt) and the Cj*czcD*^*-*^ strain was measured by ICP-MS. Cells were grown for 24 hours in MH broth with and without 25 μM Zn(II) supplementation as indicated and the Zn(II) content of mid logarithmic phase cells measured. Data are the mean of three biological replicates with standard error of the mean indicated. The * symbol indicates a p value of <0.05 from a two way ANOVA test.

To investigate the function of CzcD, it’s gene was inactivated by insertional mutagenesis in *C*. *jejuni* NCTC 11168 to create strain Cj*czcD*^*-*^ and the ability of this strain to grow in media supplemented with increasing concentrations of a range of metals examined. The Cj*czcD*^*-*^ strain was hypersensitive to Zn(II) compared to the parental wild-type (>5-fold), and to a lesser extent cobalt, but not to other metals tested (Fig 3A). Notably, Zn(II) resistance was restored to the Cj*czcD*^*-*^ strain by reintroducing a single copy of *czcD in trans* on the chromosome to create the genetically complemented Cj*czcD*^*+*^ strain (Fig 3A) and similar complementation with *czcD* site directed mutants with altered Zn(II) binding sites (S1 Fig) failed to restore Zn(II) tolerance (S4 Fig). We therefore demonstrate a primary role for CzcD in *C*. *jejuni* Zn(II) resistance. A likely consequence of loss of a Zn(II) exporter is that cells over-accumulate Zn(II). Indeed total cellular Zn(II) content of Cj*czcD*^*-*^ cells, as measured by inductively-coupled plasma mass spectroscopy (ICP-MS), was increased compared to wild-type following growth in media both with and without 25 μM Zn(II) supplementation (Fig 3B), a level which is not inhibitory to either strain (Fig 3A).

### Zn(II) and Co(II) regulation of CzcD

**Fig 4:**
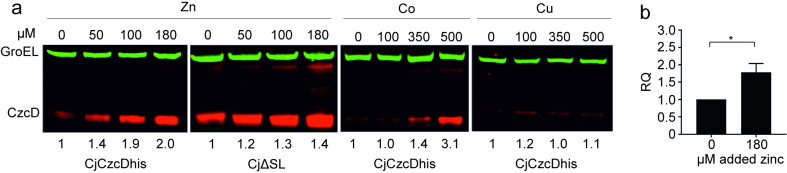
Zn(II) induction of CzcD is SL*czcD* independent. (a) The *C*. *jejuni* NCTC 11168 *czcD*his strain (CjCzcDhis) and the derivative lacking SL*czcD* (CjΔSL) were grown from an initial OD_600_ of 0.05 for 24 hours at 37°C in MH broth with Zn(II), Co(II) and copper added at the indicated concentrations. Cells were harvested and following SDS-PAGE of soluble lysates, western blots were probed with anti-GroEL (green) as a loading control and anti-histidine to detect CzcD (red). Relative CzcD signal values are presented below the blots and were standardised to the GroEL internal control signal and then normalised to the no added metal condition. These data are representative of at least three replicate experiments. (b) qRT-PCR of the *czcD* transcript for the wild type strain grown with and without added Zn(II) as indicated. The data were obtained from three biological replicates and error bars represent the calculated standard error of the RQ values of the biological repeats. A one sample t-test applied to the mean of ΔΔCt values gave a significant p value of <0.05.

Having established that CzcD is a Zn(II)/Co(II) resistance determinant we investigated whether these metals regulate CzcD production. When CjCzcDhis was grown with varying sub-inhibitory levels of Zn(II), Co(II) or copper, CzcD levels increased with added Zn(II) and Co(II) but not copper (Fig 4A), consistent with a role for CzcD in Zn(II) and Co(II) export. Both qRT-PCR (Fig 4B) and promoter reporter fusion data (S5 Fig) indicate that this is a transcriptional effect on the promoter driving *czcD* expression (Fig 1A). In other bacteria, Zn(II)-exporting CzcD proteins are typically expressed in response to Zn(II) due to the actions of one of the Zn(II)-dependent transcriptional regulators ZntR, CzrA or ScrA (and their aliases). However, whilst related sequences are present within the *C*. *jejuni* genome they lack the characteristic Zn(II)-sensing motifs (reviewed in [43]), thus providing few clues as to the mechanism of CzcD metalloregulation. The *czcD* gene is transcribed downstream of Cj1164c, encoding a Zn(II) finger-containing protein, which could potentially influence CzcD expression (Fig 1A). However, disruption of Cj1164c (by insertion of a promoterless kanamycin resistance cassette) did not abolish Zn(II) induction of CzcDhis (S6 Fig). Importantly, removal of the 75 bp Cj1164c/*czcD* intergenic region in strain CjΔSL (Fig 1A) also did not abolish Zn(II)-dependent induction of CzcDhis (Fig 4A), demonstrating that the Zn(II)-responsive expression of CzcD is independent of the RNAT.

### *C*. *jejuni* SLczcD-mediated temperature dependent Zn(II) sensitivity

**Fig 5:**
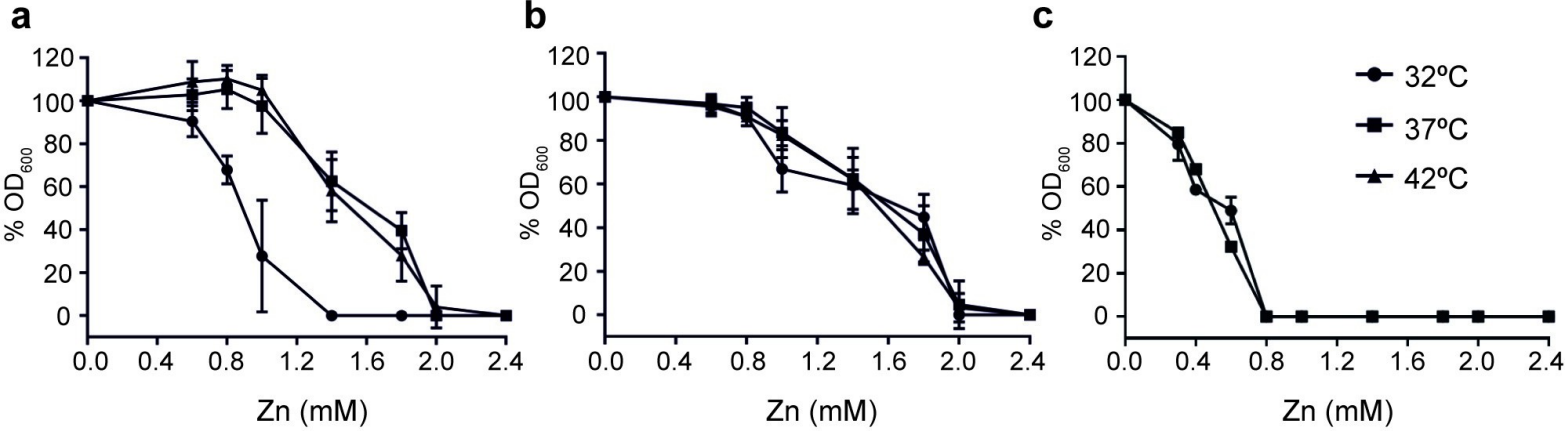
The SL*czcD* influences *C*. *jejuni* temperature dependent Zn(II) sensitivity. Following eight hours of growth in MEM-α containing varying Zn(II) concentrations and at three incubation temperatures, OD_600_ values of *C*. *jejuni* NCTC 11168 (a), a derivative lacking SL*czcD* (b) and the *czcD*^*-*^ mutant strain (c) were measured. All readings were normalised to the OD_600_ value obtained from media lacking Zn(II) and expressed as a percentage. Each data point is the average of three biological replicates each performed in technical triplicate with error bars indicating the standard error of the biological repeats.

Having demonstrated temperature-dependent CzcD production and the role of CzcD in mediating Zn(II) resistance we predicted that *C*. *jejuni* Zn(II) resistance would be increased at the elevated temperatures of a host. The ability of *C*. *jejuni* to grow in media titrated with increasing Zn(II) levels was therefore examined over a range of temperatures (32, 37 and 42°C) that support growth. Note that for these experiments a defined medium, minimal essential medium alpha (MEM-α), was used that allows *C*. *jejuni* to tolerate higher levels of Zn(II) supplementation (Fig 5A) than when grown in Mueller-Hinton (MH) medium (Fig 3A). Following 8 hours growth the OD_600_ of cultures was measured and data normalised to readings obtained without added Zn(II) (Fig 5). For the wild-type strain Zn(II) resistance was elevated at both 37 and 42°C compared to 32°C (Fig 5A). Importantly the derived CjΔSL strain was no longer more sensitive to Zn(II) at 32°C (Fig 5B) precluding a more general effect of differing growth rate of strains on zinc sensitivity. This temperature-mediated difference in Zn(II) sensitivity was also lost on inactivation of *czcD* (Fig 5C), with the Cj*czcD*^*-*^ strain showing similar Zn(II) hypersensitivity at 32 and 37°C. Combined, these findings confirm SL*czcD*-mediated temperature regulation of CzcD in *C*. *jejuni* confers increased Zn(II) resistance at host body temperatures.

### CzcD enhances killing in the *Galleria mellonella* larval infection model

**Fig 6:**
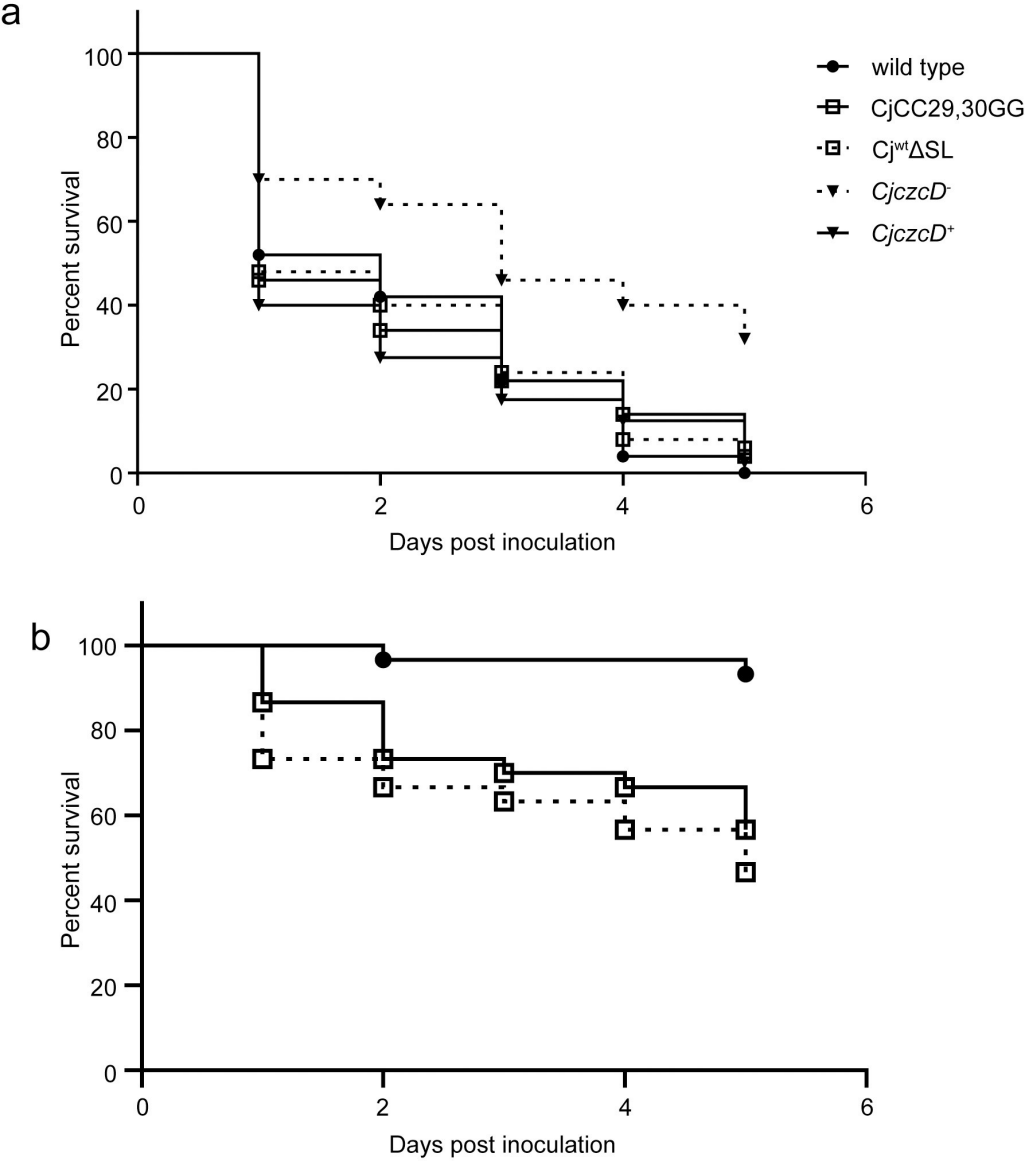
CzcD and associated temperature regulation are involved in *Galleria mellonella* larval killing. a. Strains of *C*. *jejuni* were grown in MH broth, washed twice in PBS and ~10^6^ CFU injected into *G*. *mellonella* larvae that were incubated at 37°C and scored daily for survival. Strains were wild-type NCTC 11168, *czcD* insertionally inactivated mutant (Cj*czcD*^*-*^), the derived genetically complemented strain (Cj*czcD*^*+*^), the derivative lacking SL*czcD* (Cj^wt^ΔSL) or with the derepressing mutation CC29,30GG (CjCC29,30GG). Data represent five biological replicates for all strains except Cj*czcD*^*+*^ with four biological replicates. Each biological replicate consisted of injecting ten larvae (i.e. n = 50 or 40). Pairwise p values were calculated using a Log-rank (Mantel-Cox) test implemented in GraphPad Prism. Data for the Cj*czcD*^*-*^ strain was statistically significantly different when compared to all four other strains with p values of <0.0001, <0.0001, 0.0001 and 0.0004 for the wild-type, Cj*czcD*^*+*^, CjCC29,30GG and CjΔSL comparisons, respectively. No other pairwise comparisons were statistically significant. b. Similar experiment as for (a) except *C*. *jejuni* strains were grown and larvae incubated at 32°C, and larvae were injected with ~10^7^ CFU. Data represent three biological replicates with ten larvae per experiment per strain (n = 30). P values were calculated as in (a). The results obtained for pairwise comparison of the wild-type with CjCC29,30GG and CjΔSL strains were statistically significant with p values of 0.0009 and <0.0001, respectively. There was no statistically significant difference between the CjCC29,30GG and CjΔSL strains.

We evaluated the role of CzcD using the *G*. *mellonella* larval infection model. Larvae were infected with wild-type *C*. *jejuni* NCTC 11168, the *czcD*^*-*^ and the genetically complemented *czcD*^*+*^ strain. Approximately 10^6^ colony forming units (cfu) of mid logarithmic phase MH broth grown bacteria were injected into larvae and following incubation at 37°C larvae were scored as alive or dead at daily intervals. The Cj*czcD*^*-*^ strain was attenuated compared to the parental wild-type strain and wild-type levels of larval killing were restored in the Cj*czcD*^*+*^ complemented strain (Fig 6A). These data demonstrate a role for CzcD in *C*. *jejuni* larval killing in this simple infection model and suggest that host induced Zn(II) toxicity is a feature of the innate immune response in *G*. *mellonella* larvae.

An advantage of the *G*. *mellonella* model system is that larvae can be incubated at a range of temperatures and we therefore investigated *C*. *jejuni* induced larval killing at 32°C, a temperature known to inhibit CzcD production in *C*. *jejuni* (Fig 2A). Wild-type *C*. *jejuni* and derivatives with de-repressing mutations within SL*czcD* (CjCC29,30GG) or with the SL*czcD* removed (CjΔSL) were similarly used to inoculate larvae but at a higher dose of 10^7^, rather than 10^6^ cfu, to overcome the delayed killing at this lower temperature. We have shown herein that at 32°C CzcD levels are elevated in CjCC29,30GG and CjΔSL compared to the wild-type (Fig 2A). Crucially, both the CjCC29,30GG and CjΔSL strains show enhanced killing of *G*. *mellonella* larvae compared to wild-type at 32°C (Fig 6B), but not at 37°C (Fig 6A). These data demonstrate that CzcD is involved in larval killing regulated by temperature through the activity of the SL*czcD* RNAT.

## Discussion

Several lines of evidence demonstrate a role for the *C*. *jejuni* CDF family member CzcD as a Zn(II)/Co(II) exporter: (i) its expression is induced by Zn(II) and Co(II) (Fig 4A); (ii) *C*. *jejuni* mutants with inactivated *czcD* are hypersensitive to Zn(II) and to a lesser extent Co(II) (Fig 3A); and (iii) *czcD* mutants over accumulate Zn(II) (Fig 3B). Importantly, we show that CzcD production is controlled at the post transcriptional level by an intergenic RNAT located immediately upstream of *czcD* (Figs 1 and 2), which confers Zn(II) resistance in response to the environmental cue of elevated host body temperatures (Fig 5). The RNAT allows for temperature-regulated gene expression independently of other *C*. *jejuni* factors since it is functional in a heterologous host, *E*. *coli* (Figs 1 and S3). Finally, using a Galleria infection model we show that CzcD is involved in larval killing (Fig 6A) and that disruption of the RNAT, thereby allowing CzcD production at lower temperatures, increases killing at these lower temperatures relative to the wild-type strain (Fig 6B). This is the first identified *C*. *jejuni* RNAT and the association with a Zn(II)-resistance determinant has not been previously reported in bacteria. Its role in regulating production of CzcD suggests that Zn(II) export is important during interaction of this pathogen with a host, ultimately allowing for successful colonisation.

Overcoming metal toxicity is emerging as a virulence mechanism of diverse bacterial pathogens with Zn(II) export to overcome host induced Zn(II) toxicity now an established bacterial virulence mechanism [44]. In *Helicobacter pylori*, a close relative of *C*. *jejuni*, a metal efflux pump of the resistance-nodulation-cell division (RND)-type is required for gastric colonization in a gerbil model [45]. Notably in *Streptococcus pyogenes*, CzcD was also protective against innate immune responses involving exposure of bacteria to elevated levels of Zn(II) in neutrophils [22,46]. A recent global transposon mutagenesis screen demonstrated the significance of *C*. *jejuni* metal handling systems for colonisation of a mouse intestinal infection model [23]. Mutants with insertions in the various iron uptake systems were surprisingly not colonisation defective although this may be due to redundancy, whilst insertions within genes encoding deduced copper and Zn(II) handling systems were significantly under-represented in output pools. However, for copper, despite the multi-copper oxidase CueO and Cu(I) exporter Cj1161c/CopA being required for *C*. *jejuni* copper tolerance [38] insertions within either of these genes did not significantly affect mouse colonisation whereas a colonisation defect was associated with a second predicted multi-copper oxidase. Notably, insertions within both the *znuABC* genes encoding a Zn(II) uptake system previously shown to be important for chicken colonisation [17,47] and Zn(II) resistance associated *czcD* characterised herein were significantly underrepresented in the output pools from the mouse intestine. These data suggest that both Zn(II) limitation and poisoning are encountered by *C*. *jejuni* in this model. The identification of CzcD as important for both mouse intestinal colonisation and for host killing in a larval infection model highlights the significance of countering Zn(II) toxicity in these diverse hosts and suggests a conserved Zn(II) poisoning strategy employed in both. Furthermore, in common with the regulation of key virulence factors in major pathogens, the exploitation of RNA-mediated thermosensing to regulate its expression restricts CzcD production to environments with elevated host body temperatures.

The relationship between Zn(II) and *Campylobacter* infection is therefore complex with a requirement for systems to overcome both Zn(II) deficiency and toxicity for *C*. *jejuni* host colonisation [17,47]. Moreover, Zn(II) sequestration by the host antimicrobial protein S100A12 (alias calgranulin C), which accumulates in the gastrointestinal tract of *C*. *jejuni* infected patients, is known to inhibit *C*. *jejuni* growth [48]. Yet, using a recently developed mouse model of *C*. *jejuni* infection, a Zn(II) deficient diet was also seen to promote more severe outcomes of infection including more persistent and bloody diarrhoea, increased weight loss, prolonged inflammation, increased levels of inflammatory biomarkers and altered intestinal morphology [49]. Thus, the fine-tuning of both Zn(II) uptake and Zn(II) efflux are likely to be a requirement for *C*. *jejuni* for successful host colonisation and infection, and it will be intriguing to identify where and when these opposing activities are critical. The precise coordination of Zn(II) import and export to protect against host innate immune defences is also a likely requirement for other pathogens. Notably, group A *Streptococcus* must overcome both Zn(II) starvation and toxicity during infection, with the former being encountered extracellularly and the latter upon phagocytosis by neutrophils, with Zn(II) import and export systems contributing to virulence [22].

The *czcD* gene is located within the same operon as genes Cj1162c and Cj1161c/CopA involved in copper homeostasis (Fig 1A). Cj1161c/CopA, a copper translocating P_1_-type ATPase, is involved in the export of copper from the cytoplasm to the periplasm [38] and a *C*. *jejuni copA* transposon mutant was hypoinvasive for cultured intestinal epithelial cells [50]. Furthermore, insertional inactivation of *copA* or *cueO*, also associated with copper-resistance [38], resulted in *C*. *jejuni* attenuation in the chicken colonisation model [51]. The presumed transcriptional co-regulation of Zn(II) (CzcD) and copper (Cj1161c/CopA) resistance determinants in *C*. *jejuni* suggests a response against the so-called “brass dagger” component of the innate immune response, whereby bacteria are killed through exposure to the combined toxicity of copper and Zn(II) [52]. Indeed, the combinatorial activity of copper and Zn(II) intoxication against Salmonella, with a bacterial requirement for both Zn(II) and copper export, has been demonstrated in infected macrophages [19]. Significantly we have demonstrated that the SL*czcD*, acts to regulate CzcD at the post transcriptional level, and may therefore enable a further level of differential regulation of the Zn(II) and copper resistance determinants mediated via temperature. It is tempting to speculate that *C*. *jejuni* encounters environments, presumably outside of its warm blooded hosts, where Zn(II) export is disadvantageous and yet copper export must be maintained.

The tight control of bacterial cellular Zn(II) pools is needed to ensure a supply for metabolic processes whilst avoiding Zn(II)-poisoning [8]. However, this also provides a potential vulnerability to *Campylobacter* that can be exploited for the development of antimicrobials and for novel approaches for its control in the food chain. Zn(II) has been used as an antimicrobial, for example in food packaging [53], as well as in animal (including poultry) feeds to promote growth and to restrict the proliferation of pathogenic bacteria in the gut [54,55]. Indeed, feeding piglets high dosage Zn(II) oxide causes a significant reduction in their faecal excretion of *Campylobacter coli* [56]. Clinical trials have also demonstrated the benefits of adding Zn(II) to oral rehydration solution in reducing the morbidity and mortality associated with severe diarrheal diseases in infants [57,58]. The identification of a temperature regulated *C*. *jejuni* Zn(II) resistance determinant herein potentially informs further development of strategies to exploit metal toxicity and reduce *Campylobacter* levels in food products.

A major finding of our work is that an RNAT functions in *C*. *jejuni* to regulate CzcD production. At the sequence level RNATs are poorly conserved among bacterial species, though distinct structural classes such as ROSE (repression of heat shock gene expression) and FourU elements have been proposed. ROSE elements are relatively complex with between two and four stem-loop regions and typically regulate heat shock genes [59], whilst fourU RNATs characteristically contain four contiguous uridine residues base-pairing with the AGGA SD sequence [60]. The *czcD* RNAT belongs to neither class having a relatively small structure combined with perfect Watson-Crick base pairing of the SD (AGGA) and anti-SD (UCCU) sequence (Fig 1), an arrangement found in the *Synechocystis* heat shock protein 17 RNAT [61]. Although the predicted canonical pairing between the SD and the anti-SD results in relatively high predicted thermostability (S1 Table), this may be alleviated by the large proportion of weaker A-U pairs including a stretch of five A-U pairs immediately above the RBS region and the presence of two small internal loops (A20·C35 and A15·A40) in the stem region which initiate melting of the structure in response to temperature [62–64]. Indeed, mutations that introduced complementary base pairs at these two regions had a significant repressing effect on temperature regulation in *E*. *coli* (Fig 1D). Both the stem and loop regions in the *czcD* RNAT therefore appear important in the stability and flexibility of the structure, thus enabling it to respond in *C*. *jejuni* to the small temperature shifts (32 to 42°C) reported here. The *czcD* RNAT is also unusual due to being coded for in an intergenic region of a multigene operon (Fig 1A). RNATs are generally found in the 5’ UTR of a transcript though in *Yersinia* an RNAT is located between the *yscW* and *lcrF* genes [29] in a dicistronic operon. Hence, the structure and intra-genic location of the *C*. *jejuni* RNAT in a four gene operon appear unique.

It is now clear that innate immune defences exploit the combined toxic properties of Zn(II) and copper. Here we reveal that Zn(II) resistance in *C*. *jejuni* is triggered post-transcriptionally by temperature, via an RNAT. We speculate that this novel *C*. *jejuni* RNAT provides a mechanism for the expression of the co-transcribed Zn(II) and copper exporters to be uncoupled such that Zn(II) export is prevented in the cooler environments outside of its hosts, presumably where Zn(II) is limited and Zn(II) export is disadvantageous, but is rapidly expressed in response to the environmental signal of its warm blooded hosts and where both Zn(II) and copper resistance are required.

## Materials and methods

### Bacterial strains and growth conditions

Primers, strains and plasmids are described in S2, S3 and S4 Tables respectively. *E*. *coli* were routinely grown at 37°C on Luria-Bertani (LB) agar plates or in LB broth with shaking. *C*. *jejuni* NCTC 11168 and derived strains were grown at temperatures of 32, 37 or 42°C in a Variable Atmosphere Incubator (Don Whitley Ltd.) with gas settings of 5% O_2_, 10% CO_2_ and 85% N_2_. Strains were maintained on Mueller-Hinton (MH) or Columbia blood agar (CBA) plates, and growth experiments carried out in either MH broth or defined minimal essential medium alpha (MEM-α) with no glutamine or phenol red (Gibco) and supplemented with 20 mM serine and pyruvate. Media were supplemented where appropriate with antibiotics at the following concentrations: ampicillin (100 μg/ml), kanamycin (50 μg/ml), and chloramphenicol (17 μg/ml for *C*. *jejuni* or 34 μg/ml for *E*. *coli*).

### Inactivation of *czcD* and genetic complementation

The *czcD* gene was inactivated by insertion of a kanamycin resistance cassette lacking a terminator with the same transcriptional polarity. Approximately 550 bp PCR amplicons, containing 5’ and 3’ regions of *czcD*, were produced using primer pairs Cj1163-UF/Cj1163-UR and Cj1163-DF/Cj1163-DR, with HindIII sites incorporated within Cj1163-UR and Cj1163-DF. The two products were joined together in an overlap PCR reaction and ligated to pGEM-T Easy (Promega) to create pGEMCj1163c. The kanamycin resistance cassette was ligated into the HindIII site of pGEMCj1163c, immediately following base 322 of the *czcD* sequence, and the resulting construct introduced into *C*. *jejuni* NCTC 11168 by electroporation. Mutants were selected on CBA plates supplemented with kanamycin and interruption of *czcD* by insertion of the kanamycin resistance cassette verified by PCR and sequencing.

For complementation of the *czcD* mutant, a linear DNA fragment was generated by the gene Splicing by Overlap Extension (SOEing) method (as outlined in [65]), comprising the Cj0223c pseudogene sequence flanking a chloramphenicol resistance cassette and *czcD*. Briefly three PCR products were generated (i) 1313 bp containing Cj0223 5’ sequences and the chloramphenicol resistance cassette with associated promoter using primers 0223upF and endcatR with pCJC1 [66] as template, (ii) 726 bp containing *czcD* using primers Cj1163compF and Cj1163compR with *C*. *jejuni* NCTC 11168 genomic DNA as template, and (iii) 973 bp containing Cj0223 3’ sequences using primers 0223downf and 0223downR with pCJC1 as template. Following purification, 50, 90 and 50 ng, respectively, of the products were used with Phusion High-fidelity PCR master mix (New England Biolabs) in a reaction with primers 1633F and 1636R at a final concentration of 0.06 μM to generate the full length product (3.012 kb), using the following conditions: 1 cycle of 98°C for 3 minutes, 61°C for 1 minute, 72°C for 10 minutes, 98°C for 1 minute; 40 cycles of 98°C for 15 seconds, 55°C for 20 seconds and 72°C for 1 minute; followed by a 10 minute final extension at 72°C. The resulting PCR product, with the chloramphenicol resistance cassette and associated promoter located immediately upstream of *czcD* and in the same transcriptional orientation so that it can drive *czcD* expression, was introduced into the *czcD*^*-*^ strain by electroporation and transformants selected on chloramphenicol-containing CBA.

### Construction of *C*. *jejuni* strains expressing histidine-tagged CzcD and variant Cj1164c/*czcD* intergenic regions

A ligase cycling reaction (LCR) was used to assemble DNA fragments, using single-stranded synthetic bridging oligonucleotides as described by [67], to generate a *C*. *jejuni* strain that produces a C-terminal hexa-histidine tagged CzcD (CjCzcDhis) from the native locus. Two DNA fragments were PCR amplified using *C*. *jejuni* NCTC 11168 genomic DNA as template: (i) containing *czcD* with six histidine codons (cat cac cat cac cat cac) at the 3’ end plus 545 bp of *czcD* upstream sequences using primers LCR_A F and LCR_B R, and (ii) 558-bp of *czcD* downstream sequences using primers LCR_C F and LCR_C R. The fragments were 5′-phosphorylated before assembly, using T4 polynucleotide kinase in reactions containing 90 fmol of each DNA fragment, 5 mM ATP, 10 U T4 polynucleotide kinase, and 1× Ampligase reaction buffer (Epicenter Biotechnologies, Madison, WI) for 1 hour at 37°C followed by 20 minutes incubation at 65°C. Ligase cycling reactions were subsequently performed with 7.5 U Ampligase thermostable DNA ligase, 3 nM of each phosphorylated DNA fragment, and 30 nM of the bridging oligonucleotide bridge_BC with 1 cycle of 94°C for 2 minutes followed by 50 cycles of 94°C for 10 seconds, 55°C for 30 seconds, and 66°C for 60 seconds. The resulting fragment, containing hexa histidine-tagged *czcD* flanked by wild-type 5’ and 3’ sequences, was introduced into the Zn(II)-sensitive Cj*czcD*^*-*^ strain by electroporation and mutants selected on CBA supplemented with 300 μM ZnSO_4_ (a level that is inhibitory for Cj*czcD*^-^). Integration of the DNA fragment and replacement of the kanamycin resistance cassette was confirmed by PCR and sequencing. The resulting strain is essentially wild-type other than the introduction of six histidine codons at the 3’ end of *czcD* to code for CzcDhis.

A derivative of CjCzcDhis with de-repressing mutations (CC29,30GG) in the SL*czcD* was generated in the same way but assembled from three fragments: (i) 545 bp of *czcD* upstream sequences amplified using primers LCR_A F and LCR_A R, the latter including a CC29,30GG substitution within SL*czcD*, (ii) containing hexa-histidine tagged *czcD* amplified using primers LCR_B F and LCR_B R, and (iii) 558 bp of *czcD* downstream sequences using primers LCR_C F and LCR_C R, and using the bridging oligonucleotide bridge_AB.

Two further derivatives lacking SL*czcD*, in the CzcDhis producing strain and in wild-type NCTC 11168, were similarly generated but using the SOEing method. Two PCR products were generated using *C*. *jejuni* containing *czcD*-his or wild-type NCTC 11168 genomic DNA as template: (i) 511 bp containing SL*czcD* 5’ sequences using primers LCR_A F and deltaSL1, and (ii) 1512 bp containing SL*czcD* 3’ sequences including hexa-histidine tagged *czcD* and downstream sequences using primers deltaSL2 and LCR_C R. Following purification, the two PCR products were mixed in a 1:1 ratio and used as template in Phusion High-Fidelity PCR to generate the full length 2.013 kb product with primers LCR_A F and LCR_C R (0.06 μM final concentration), using the following conditions: 1 cycle of 98°C for 3 minutes, 61°C for 1 minute, 72°C for 10 minutes, 98°C for 1 minute; and 40 cycles of 98°C for 15 seconds, 65°C for 20 seconds and 72°C for 1 minute; followed by a 10 minute final extension at 72°C. The final product was ligated to pGEM-T Easy and the resulting construct introduced into the *czcD*^*-*^ strain of *C*. *jejuni* by electroporation and mutants selected on CBA supplemented with 300 μM ZnSO_4_ as above.

### Determination of metal tolerance and Zn(II) quotas

*C*. *jejuni* strains were grown on MH agar plates, sub-cultured into MH broth and following overnight growth diluted to an OD_600_ of 0.05 in MH broth or MEM-α supplemented with varying levels of metals (5 ml cultures in wells of six well tissue culture plates). Growth was monitored by measuring OD_600_. Metal quotas of cells were determined essentially as described previously [68], but using cells washed in Dulbecco’s PBS without CaCl_2_ and MgCl_2_ for cell culture (Sigma-Aldrich) and expressed as atoms per absorbance unit at 595 nm (determined using a synergy HT plate reader, BioTek Instruments Inc.). All assays were performed in triplicate on at least three separate occasions.

### SDS-PAGE and Western blot analyses

Western blot analyses were performed using whole cell lysates of *E*. *coli* and *C*. *jejuni* grown as described in individual experiments. Cells were harvested by centrifugation, suspended in PBS to a standardised OD_600_ and LDS loading buffer (Thermo Fisher Scientific) added. Samples were incubated at 95°C for 5 minutes, centrifuged at 12,000 g for 5 minutes and electrophoresed for 90 minutes at 130 V in 10% NuPAGE Bis-Tris gels (Invitrogen) using NuPAGE MES (2-(N-morpholino)ethanesulfonic acid) SDS Running Buffer (Invitrogen). Proteins were transferred to nitrocellulose membrane using an XCell blot module (Invitrogen) in Tris-Glycine transfer buffer [192 mM glycine, 25 mM Tris pH 8.3, 20% (v/v) methanol, 0.01% (w/v) SDS] at 25 V for 90 minutes. The membrane was blocked with 3% (w/v) BSA in PBS overnight at 4°C, washed twice in PBS with 0.1% (v/v) Tween 20 and once in PBS for 5 minutes. For detection of histidine-tagged proteins, the membrane was incubated for one hour at room temperature with anti-histidine primary antibody (Qiagen), diluted 1:1000 in PBS, followed by a one hour incubation with IRDye 680RD goat anti-mouse IgG (LiCor Biosciences, Lincoln USA) as a secondary antibody. For detection of GFP, rabbit-derived anti-GFP antibody (Invitrogen) diluted 1:2000 in PBS and IRDye 800CW goat-derived anti-rabbit IgG (LiCor Biosciences) were used. For detection of maltose-binding protein (MBP), murine anti-MBP antibody (New England Biolabs) diluted 1:10,000 was used as primary antibody followed by IRDye 680RD goat anti-mouse IgG (LiCor Biosciences). Anti-GroEL and anti-RecA antibodies (Abcam) were diluted 1:1000. After washing membranes in PBS, signals were detected using Odyssey CLx imaging system (Li-Cor Biosciences) and signals quantified using Image Studio software (LiCor Biosciences).

### Construction of the *malE*/*gfp* and *lacZ E*. *coli* reporter plasmids and derivatives

A reporter construct was generated which contained SL*czcD* and the *czcD* start codon placed between the two reporter genes *malE* and *gfp* such that *gfp* was placed in the same position as *czcD* with respect to SL*czcD* on the *C*. *jejuni* NCTC 11168 chromosome. The *gfp*^*TCD*^ gene [69] was ligated into NdeI/SalI digested pMAL-c5x (New England Biolabs) downstream of *malE* to create pMG. A DNA fragment containing SL*czcD* and the *czcD* start codon (plus 7 nucleotides at the 5′ end that contain a stop codon and an NdeI-compatible overhang, and an NcoI-compatible overhang at the 3′ end) was constructed by annealing two single-stranded complementary synthetic oligonucleotides SL1 and SL2 (S2 Table). These were incubated at equimolar concentrations in annealing buffer (10 mM Tris, 1 mM EDTA, 50 mM NaCl, pH 8.0) at 95°C for 5 minutes and cooled to room temperature. The annealed fragment was then ligated into NdeI/NcoI digested pMG (note the NcoI site is at the 5’end of the introduced *gfp*^*TCD*^) and the resultant construct, pMSLG, was confirmed by PCR and sequencing. Q5 Site-Directed Mutagenesis (New England Biolabs) was used to generate pMSLG with the following mutations within SL*czcD* (refer to Fig 1 for numbering): CC29,30GG; CC29,30UU; A36U; A41G; G59C; U34A; G59C,C38G; and U34A,A63U; plus a derivative lacking residues 22 to 45 of the SL*czcD* (designated pMSLG^*ΔSL*^).

To create a second *lacZ* based reporter system a PCR amplified *lacZ* gene (primer pair lacZNheIF and laczXbaIR—S2 Table) was directionally cloned into plasmid pMLBAD using NheI and XbaI sites to create pBAD*lacZ*. The SL*czcD* with NcoI and NdeI termini was ligated into pBAD*lacZ* immediately upstream of the NdeI creating pSL*lacZ*. In this construct transcription of the stem-loop regulated reporter *lacZ* is driven by the arabinose-inducible and glucose-repressible P_BAD_ promoter. Plasmid pSL*lacZ* derivatives pSL*lacZ*A41G and pSL*lacZ*CC29,30GG were created by site directed mutagenesis. Strains of *E*. *coli* containing each plasmid were grown to mid-log phase at 37°C, and induced with arabinose at 30, 37, or 42°C for two hours. Cells were harvested by centrifugation, re-suspended in Z buffer (0.06 M Na_2_HPO_4_·7H_2_O, 0.04 M NaH_2_PO_4_·H_2_O, 0.01 M KCl, 0.001 M MgSO_4_, 0.27% [v/v] β-mercaptoethanol [Sigma-Aldrich]) and permeabilised with 100 μl of chloroform (Sigma-Aldrich) and 50 μl of 0.1% (w/v) sodium dodecyl sulphate (Sigma-Aldrich) per ml of cell suspension. Following addition of ortho-nitrophenyl-β-galactoside reactions were incubated at 28°C until coloured products were visible and Na_2_CO_3_ added. Absorbance values at 420 nm were used to calculate β-galactosidase activity in Miller units.

### Construction and analysis of *C*. *jejuni* GFP reporter strains

The pCJC1 plasmid [66] was modified by introduction of the Cj1164c associated promoter with *gfp* placed immediately downstream. These were inserted upstream of the chloramphenicol resistance cassette (*cat*) to create plasmid pCJC1P*gfp*. This was electroporated into *C*. *jejuni* NCTC 11168 and recombinants selected on chloramphenicol-containing media. Insertion of the Cj1164c promoter-*gfp*-*cat* cassette within the pseudogene Cj0223c to create strain CjP*gfp* was verified by PCR. Following growth of this strain in MEM-α shaking at 37°C for 24 hours, cells were pelleted by centrifugation, suspended in PBS to an OD_600_ of 1.0 and 200 μl transferred in triplicate into a black-walled 96-well plate with a transparent base. Relative fluorescence units (RFU) with excitation at 485/20 nm and emission at 528/20 nm and OD_600_ were measured in a Bio-Tek Synergy HT plate reader.

### RNA purification and quantitative RT-PCR

Mid-logarithmic *C*. *jejuni* cultures were mixed with RNA Protect Reagent (Qiagen) before total RNA extraction and purification using the RNeasy Mini Kit (Qiagen) following the manufacturer’s instructions. Samples were treated with 2.5 μl of DNase I for one hour at 37°C to eliminate DNA contamination. Reverse transcriptase reactions were carried out on 1 μg total RNA using a QuantiTect Reverse transcription kit (Qiagen) according to manufacturer’s instructions. cDNA samples were diluted 1:3 in RNase free water and quantitative RT-PCR was performed using SYBR Green I (Applied Biosystems). The PCR mix consisted of 4 μl of cDNA sample, 238 nM of forward and reverse primers, and 4 μl of Fast SYBR Green Master Mix. Each reaction contained four technical repeats and was carried out in an Applied Biosystems StepOnePlus Real-Time PCR system. RT-negative controls were included for all experiments. The *rpoA* gene was used as an internal control. Amplification conditions were initial denaturation at 95°C for 20 seconds, followed by 40 cycles of 95°C for 3 seconds and 60°C for 30 seconds. Similar primer pair efficiencies were ensured by performing a standard curve with serially diluted DNA. Relative gene expression values were calculated using the Livak method [70].

### Galleria infection

Following growth in MH broth to an OD_600_ of between 0.4 and 0.5 (mid-log growth phase), *C*. *jejuni* cells were pelleted by centrifugation, washed twice and resuspended to an OD_600_ of 1.0 in sterile PBS. For each replicate experiment ten larvae of *Galleria mellonella* (Livefoods Direct Ltd. Sheffield, UK) were injected using a 0.5 ml 29G 12.7 mm insulin syringe (VWR International Ltd.) with 10 μl of cell suspension through the rear proleg. Each experiment included a control batch of larvae injected with PBS.

## Supporting information

S1 Fig Amino acid sequence alignment of *C*. *jejuni* CzcD and *E*. *coli* YiiP. The four active site residues for metal transport in YiiP [41] are highlighted in blue along with aligned conserved CzcD residues H73, D77, H179 and D183. Alignment was produced using Clustal Omega.
(DOCX)

S2 Fig Multiple sequence alignment and mfold analysis of the Cj1164c/*czcD* intergenic region from 1626 *C*. *jejuni* strains. (a) Clustal generated multiple sequence alignment of the Cj1164c/*czcD* intergenic region including Cj1164c stop codon and *czcD* start codon. The 38 sequence variants are numbered and presented in ranked order with the number of strains with that particular sequence indicated in brackets. The NCTC 11168 sequence belongs to the most common variant 1. (b) Predicted secondary structures for all 38 sequence variants of the Cj1164c/*czcD* intergenic region from 1626 *C*. *jejuni* strains using Mfold with standard parameters.
(DOCX)

S3 Fig Thermoregulation of β-galactosidase activity mediated by the *C*. *jejuni* SL*czcD*. Strains of *E*. *coli* containing plasmids pSL*lacZ*, pSL*lacZ*A41G or pSL*lacZ*CC29,30GG as indicated were grown to mid log phase and expression of *lacZ* induced for two hours. Cells were harvested and β-galactosidase activities measured. Data presented are the mean of three (pSL*lacZ*), five (pSL*lacZ*A41G) or two (pSL*lacZ*CC29,30GG) biological repeats. Error bars represent standard error of the mean with * indicating a t-test p value of <0.05.
(TIF)

S4 Fig *C*. *jejuni* CzcD active site residue mutants do not restore Zn(II) tolerance. The Cj*czcD*^*-*^ strain was genetically complemented with wild-type *czcD* (Cj*czcD*^*+*^) and with two mutant versions each with two alanine substitutions in the Zn(II) binding active site as identified in S1 Fig. Strains were grown in MEM-α containing varying Zn(II) concentrations (in 24 well tissue culture plates) from an initial OD_600_ of 0.05 at 37°C for 24 hours and the final OD_600_ recorded. Data points are the mean of three biological repeats, each with three technical repeats and the error bars indicate standard error of the mean of three biological repeats.
(TIF)

S5 Fig In *C*. *jejuni* Zn(II) and Co(II) induce expression from the promoter upstream of Cj1164c. a. Diagrammatic representation of the region introduced onto the *C*. *jejuni* NCTC 11168 chromosome within the pseudogene Cj0223c to create strain CjP*gfp*. The arrowhead represents the σ^70^ promoter identified by RNAseq analyses [36,37] directing expression of Cj1164c to Cj1161c. The green arrow is the *gfp* reporter under control of the promoter and *cat* is a chloramphenicol resistance cassette. b. Cells of *C*. *jejuni* strain CjP*gfp* were grown for 24 hours in MEM-α media (-) or in MEM-α supplemented with sub-inhibitory concentrations of Zn(II) - 1.8 mM, Co (II) - 0.4 mM, or copper—0.4 mM as indicated. Relative fluorescence was calculated by dividing the fluorescence units by the optical density measured at 600 nm. Data presented are the average of three biological repeats in technical triplicate. Error bars represent the calculated standard error of the biological repeats and p values were calculated using the one-way ANOVA with * indicating p <0.005.
(TIF)

S6 Fig The *Cj1164c* gene product is not required for CzcD induction by Zn(II). The CjCzcDhis strain and a derivative with a disrupted *Cj1164c* gene (Cj*Cj1164*^*-*^) were grown for 24 hours in MH broth with Zn(II) added at the indicated concentrations. Cells were harvested and following Western blotting of whole cell extracts, membranes were probed with anti-GroEL (green) as loading control and anti-histidine to detect CzcDhis (red). These data are representative of at least three replicate experiments.
(TIF)

S1 Table Minimum free energy values for the Cj1164c/czcD intergenic region transcript and site directed mutants characterized herein. (DOCX)

S2 Table Oligonucleotides used in this study. (DOCX)

S3 Table Strains used in this study. (DOCX)

S4 Table Plasmids used in this study. (DOCX)

## References

[1] Kirk MD, Pires SM, Black RE, Caipo M, Crump JA, Devleesschauwer B, et al World Health Organization Estimates of the Global and Regional Disease Burden of 22 Foodborne Bacterial, Protozoal, and Viral Diseases, 2010: A Data Synthesis. PLoS Med. 2015;12(12):e1001921 10.1371/journal.pmed.1001921 26633831 PMC4668831

[2] Platts-Mills JA, Kosek M. Update on the burden of Campylobacter in developing countries. Curr Opin Infect Dis. 2014;27(5):444–50. 10.1097/QCO.0000000000000091 25023741 PMC4542018

[3] Burnham PM, Hendrixson DR. Campylobacter jejuni: collective components promoting a successful enteric lifestyle. Nat Rev Microbiol. 2018;16(9):551–65. 10.1038/s41579-018-0037-9 29892020

[4] Goodfellow JA, Willison HJ. Guillain-Barré syndrome: a century of progress. Nat Rev Neurol. 2016;12(12):723–31. 10.1038/nrneurol.2016.172 27857121

[5] Harrer A, Bücker R, Boehm M, Zarzecka U, Tegtmeyer N, Sticht H, et al Campylobacter jejuni enters gut epithelial cells and impairs intestinal barrier function through cleavage of occludin by serine protease HtrA. Gut Pathog. 2019;11:4 10.1186/s13099-019-0283-z 30805031 PMC6373145

[6] Watson RO, Galán JE. Campylobacter jejuni survives within epithelial cells by avoiding delivery to lysosomes. PLoS Pathog. 2008;4(1):e14 10.1371/journal.ppat.0040014 18225954 PMC2323279

[7] Wooldridge KG, Ketley JM. Campylobacter-host cell interactions. Trends Microbiol. 1997;5(3):96–102. 10.1016/S0966-842X(97)01004-4 9080607

[8] Waldron KJ, Robinson NJ. How do bacterial cells ensure that metalloproteins get the correct metal? Nat Rev Microbiol. 2009;7(1):25–35. 10.1038/nrmicro2057 19079350

[9] Djoko KY, Ong CL, Walker MJ, McEwan AG. The Role of Copper and Zinc Toxicity in Innate Immune Defense against Bacterial Pathogens. J Biol Chem. 2015;290(31):18954–61. 10.1074/jbc.R115.647099 26055706 PMC4521016

[10] Lopez CA, Skaar EP. The Impact of Dietary Transition Metals on Host-Bacterial Interactions. Cell Host Microbe. 2018;23(6):737–48. 10.1016/j.chom.2018.05.008 29902439 PMC6007885

[11] Sheldon JR, Skaar EP. Metals as phagocyte antimicrobial effectors. Curr Opin Immunol. 2019;60:1–9. 10.1016/j.coi.2019.04.002 31063946 PMC6800623

[12] Liuzzi JP, Lichten LA, Rivera S, Blanchard RK, Aydemir TB, Knutson MD, et al Interleukin-6 regulates the zinc transporter Zip14 in liver and contributes to the hypozincemia of the acute-phase response. Proc Natl Acad Sci U S A. 2005;102(19):6843–8. 10.1073/pnas.0502257102 15863613 PMC1100791

[13] Zackular JP, Chazin WJ, Skaar EP. Nutritional Immunity: S100 Proteins at the Host-Pathogen Interface. J Biol Chem. 2015;290(31):18991–8. 10.1074/jbc.R115.645085 26055713 PMC4521021

[14] Liu JZ, Jellbauer S, Poe AJ, Ton V, Pesciaroli M, Kehl-Fie TE et al Zn(II) sequestration by the neutrophil protein calprotectin enhances Salmonella growth in the inflamed gut. Cell Host Microbe 2012;11:227–39. 10.1016/j.chom.2012.01.017 22423963 PMC3308348

[15] Hesse LE, Lonergan ZR, Beavers WN, Skaar EP. The Acinetobacter baumannii Znu system overcomes host-imposed nutrient Zn(II) limitation. Infect Immun 2019;87:e00746–19. 10.1128/IAI.00746-19 PMC6867866 31548324

[16] Zackular JP, Knippel RJ, Lopez CA, Beavers WN, Maxwell CN, Chazin WJ, Skaar EP. ZupT facilitates Clostridioides difficile resistance to host-mediated nutritional immunity. mSphere 2020;5:e00061–20. 10.1128/mSphere.00061-20 32161145 PMC7067591

[17] Gielda LM, DiRita VJ. Zinc competition among the intestinal microbiota. mBio. 2012;3(4):e00171–12. 10.1128/mBio.00171-12 22851657 PMC3419517

[18] Botella H, Peyron P, Levillain F, Poincloux R, Poquet Y, Brandli I, et al Mycobacterial p(1)-type ATPases mediate resistance to zinc poisoning in human macrophages. Cell Host Microbe. 2011;10(3):248–59. 10.1016/j.chom.2011.08.006 21925112 PMC3221041

[19] Kapetanovic R, Bokil NJ, Achard ME, Ong CL, Peters KM, Stocks CJ, et al Salmonella employs multiple mechanisms to subvert the TLR-inducible zinc-mediated antimicrobial response of human macrophages. FASEB J. 2016;30(5):1901–12. 10.1096/fj.201500061 26839376

[20] McDevitt CA, Ogunniyi AD, Valkov E, Lawrence MC, Kobe B, McEwan AG, et al A molecular mechanism for bacterial susceptibility to zinc. PLoS Pathog. 2011;7(11):e1002357 10.1371/journal.ppat.1002357 22072971 PMC3207923

[21] Eijkelkamp BA, Morey JR, Neville SL, Tan A, Pederick VG, Cole N et al, (2019). Dietary Zn(II) and the control of Streptococcus pneumoniae infection. PLoS Pathog 15:e1007957 10.1371/journal.ppat.1007957 31437249 PMC6705770

[22] Ong CY, Berking O, Walker MJ, McEwan AG. New Insights into the Role of Zinc Acquisition and Zinc Tolerance in Group A Streptococcal Infection. Infect Immun. 2018;86(6). 10.1128/IAI.00048-18 PMC5964520 29581188

[23] Gao B, Vorwerk H, Huber C, Lara-Tejero M, Mohr J, Goodman AL, et al Metabolic and fitness determinants for in vitro growth and intestinal colonization of the bacterial pathogen Campylobacter jejuni. PLoS Biol. 2017;15(5):e2001390 10.1371/journal.pbio.2001390 28542173 PMC5438104

[24] Kortmann J, Narberhaus F. Bacterial RNA thermometers: molecular zippers and switches. Nat Rev Microbiol. 2012;10(4):255–65. 10.1038/nrmicro2730 22421878

[25] Loh E, Righetti F, Eichner H, Twittenhoff C, Narberhaus F. RNA Thermometers in Bacterial Pathogens. Microbiol Spectr. 2018;6(2). 10.1128/microbiolspec.rwr-0012-2017 PMC11633587 29623874

[26] Johansson J, Mandin P, Renzoni A, Chiaruttini C, Springer M, Cossart P. An RNA thermosensor controls expression of virulence genes in Listeria monocytogenes. Cell. 2002;110(5):551–61. 10.1016/s0092-8674(02)00905-4 12230973

[27] Weber GG, Kortmann J, Narberhaus F, Klose KE. RNA thermometer controls temperature-dependent virulence factor expression in Vibrio cholerae. Proc Natl Acad Sci U S A. 2014;111(39):14241–6. 10.1073/pnas.1411570111 25228776 PMC4191814

[28] Grosso-Becerra MV, Croda-García G, Merino E, Servín-González L, Mojica-Espinosa R, Soberón-Chávez G. Regulation of Pseudomonas aeruginosa virulence factors by two novel RNA thermometers. Proc Natl Acad Sci U S A. 2014;111(43):15562–7. 10.1073/pnas.1402536111 25313031 PMC4217398

[29] Böhme K, Steinmann R, Kortmann J, Seekircher S, Heroven AK, Berger E, et al Concerted actions of a thermo-labile regulator and a unique intergenic RNA thermosensor control Yersinia virulence. PLoS Pathog. 2012;8(2):e1002518 10.1371/journal.ppat.1002518 22359501 PMC3280987

[30] Loh E, Kugelberg E, Tracy A, Zhang Q, Gollan B, Ewles H, et al Temperature triggers immune evasion by Neisseria meningitidis. Nature. 2013;502(7470):237–40. 10.1038/nature12616 24067614 PMC3836223

[31] Loh E, Lavender H, Tan F, Tracy A, Tang CM. Thermoregulation of Meningococcal fHbp, an Important Virulence Factor and Vaccine Antigen, Is Mediated by Anti-ribosomal Binding Site Sequences in the Open Reading Frame. PLoS Pathog. 2016;12(8):e1005794 10.1371/journal.ppat.1005794 27560142 PMC4999090

[32] Matsunaga J, Schlax PJ, Haake DA. Role for cis-acting RNA sequences in the temperature-dependent expression of the multiadhesive lig proteins in Leptospira interrogans. J Bacteriol. 2013;195(22):5092–101. 10.1128/JB.00663-13 24013626 PMC3811586

[33] Twittenhoff C, Heroven AK, Mühlen S, Dersch P, Narberhaus F. An RNA thermometer dictates production of a secreted bacterial toxin. PLoS Pathog. 2020;16(1):e1008184. 10.1371/journal.ppat.1008184 PMC6992388 31951643

[34] Kouse AB, Righetti F, Kortmann J, Narberhaus F, Murphy ER. RNA-mediated thermoregulation of iron-acquisition genes in Shigella dysenteriae and pathogenic Escherichia coli. PLoS One. 2013;8(5):e63781 10.1371/journal.pone.0063781 23704938 PMC3660397

[35] Wei Y, Kouse AB, Murphy ER. Transcriptional and posttranscriptional regulation of Shigella shuT in response to host-associated iron availability and temperature. Microbiologyopen. 2017;6(3). 10.1002/mbo3.442 PMC5458455 28127899

[36] Dugar G, Herbig A, Förstner KU, Heidrich N, Reinhardt R, Nieselt K, et al High-resolution transcriptome maps reveal strain-specific regulatory features of multiple Campylobacter jejuni isolates. PLoS Genet. 2013;9(5):e1003495 10.1371/journal.pgen.1003495 23696746 PMC3656092

[37] Porcelli I, Reuter M, Pearson BM, Wilhelm T, van Vliet AH. Parallel evolution of genome structure and transcriptional landscape in the Epsilonproteobacteria. BMC Genomics. 2013;14:616 10.1186/1471-2164-14-616 24028687 PMC3847290

[38] Hall SJ, Hitchcock A, Butler CS, Kelly DJ. A Multicopper oxidase (Cj1516) and a CopA homologue (Cj1161) are major components of the copper homeostasis system of Campylobacter jejuni. J Bacteriol. 2008;190(24):8075–85. 10.1128/JB.00821-08 18931123 PMC2593206

[39] Gundogdu O, Bentley SD, Holden MT, Parkhill J, Dorrell N, Wren BW. Re-annotation and re-analysis of the Campylobacter jejuni NCTC11168 genome sequence. BMC Genomics. 2007;8:162 10.1186/1471-2164-8-162 17565669 PMC1899501

[40] Kolaj-Robin O, Russell D, Hayes KA, Pembroke JT, Soulimane T. Cation Diffusion Facilitator family: Structure and function. FEBS Lett. 2015;589(12):1283–95. 10.1016/j.febslet.2015.04.007 25896018

[41] Lu M, Fu D. Structure of the zinc transporter YiiP. Science. 2007;317(5845):1746–8. 10.1126/science.1143748 17717154

[42] Zuker M. Mfold web server for nucleic acid folding and hybridization prediction. Nucleic Acids Res. 2003;31(13):3406–15. 10.1093/nar/gkg595 12824337 PMC169194

[43] Capdevila DA, Edmonds KA, Giedroc DP. Metallochaperones and metalloregulation in bacteria. Essays Biochem. 2017;61(2):177–200. 10.1042/EBC20160076 28487396 PMC5858914

[44] Palmer LD, Skaar EP. Transition Metals and Virulence in Bacteria. Annu Rev Genet. 2016;50:67–91. 10.1146/annurev-genet-120215-035146 27617971 PMC5125913

[45] Stähler FN, Odenbreit S, Haas R, Wilrich J, Van Vliet AH, Kusters JG, et al The novel Helicobacter pylori CznABC metal efflux pump is required for cadmium, zinc, and nickel resistance, urease modulation, and gastric colonization. Infect Immun. 2006;74(7):3845–52. 10.1128/IAI.02025-05 16790756 PMC1489693

[46] Ong CL, Gillen CM, Barnett TC, Walker MJ, McEwan AG. An antimicrobial role for zinc in innate immune defense against group A streptococcus. J Infect Dis. 2014;209(10):1500–8. 10.1093/infdis/jiu053 24449444

[47] Davis LM, Kakuda T, DiRita VJ. A Campylobacter jejuni znuA orthologue is essential for growth in low-zinc environments and chick colonization. J Bacteriol. 2009;191(5):1631–40. 10.1128/JB.01394-08 19103921 PMC2648198

[48] Shank JM, Kelley BR, Jackson JW, Tweedie JL, Franklin D, Damo SM, et al The Host Antimicrobial Protein Calgranulin C Participates in the Control of Campylobacter jejuni Growth via Zinc Sequestration. Infect Immun. 2018;86(6). 10.1128/IAI.00234-18 PMC5964530 29610259

[49] Giallourou N, Medlock GL, Bolick DT, Medeiros PH, Ledwaba SE, Kolling GL, et al A novel mouse model of Campylobacter jejuni enteropathy and diarrhea. PLoS Pathog. 2018;14(3):e1007083 10.1371/journal.ppat.1007083 29791507 PMC5988333

[50] Novik V, Hofreuter D, Galán JE. Identification of Campylobacter jejuni genes involved in its interaction with epithelial cells. Infect Immun. 2010;78(8):3540–53. 10.1128/IAI.00109-10 20515930 PMC2916286

[51] Gardner SP, Olson JW. Interaction of Copper Toxicity and Oxidative Stress in Campylobacter jejuni. J Bacteriol. 2018;200(21). 10.1128/JB.00208-18 PMC6182239 30150230

[52] German N, Doyscher D, Rensing C. Bacterial killing in macrophages and amoeba: do they all use a brass dagger? Future Microbiol. 2013;8(10):1257–64. 10.2217/fmb.13.100 24059917

[53] Singh S, Ho Lee M, Park L, Shin Y, Lee YS. Antimicrobial seafood packaging: a review. J Food Sci Technol. 2016;53(6):2505–18. 10.1007/s13197-016-2216-x 27478206 PMC4951407

[54] Holman DB, Chénier MR. Antimicrobial use in swine production and its effect on the swine gut microbiota and antimicrobial resistance. Can J Microbiol. 2015;61(11):785–98. 10.1139/cjm-2015-0239 26414105

[55] Park SY, Birkhold SG, Kubena LF, Nisbet DJ, Ricke SC. Review on the role of dietary zinc in poultry nutrition, immunity, and reproduction. Biol Trace Elem Res. 2004;101(2):147–63. 10.1385/BTER:101:2:147 15557678

[56] Bratz K, Gölz G, Riedel C, Janczyk P, Nöckler K, Alter T. Inhibitory effect of high-dosage zinc oxide dietary supplementation on Campylobacter coli excretion in weaned piglets. J Appl Microbiol. 2013;115(5):1194–202. 10.1111/jam.12307 23869938

[57] Bhandari N, Mazumder S, Taneja S, Dube B, Agarwal RC, Mahalanabis D, et al Effectiveness of zinc supplementation plus oral rehydration salts compared with oral rehydration salts alone as a treatment for acute diarrhea in a primary care setting: a cluster randomized trial. Pediatrics. 2008;121(5):e1279–85. 10.1542/peds.2007-1939 18450870

[58] Lukacik M, Thomas RL, Aranda JV. A meta-analysis of the effects of oral zinc in the treatment of acute and persistent diarrhea. Pediatrics. 2008;121(2):326–36. 10.1542/peds.2007-0921 18245424

[59] Waldminghaus T, Gaubig LC, Klinkert B, Narberhaus F. The Escherichia coli ibpA thermometer is comprised of stable and unstable structural elements. RNA Biol. 2009;6(4):455–63. 10.4161/rna.6.4.9014 19535917

[60] Waldminghaus T, Heidrich N, Brantl S, Narberhaus F. FourU: a novel type of RNA thermometer in Salmonella. Mol Microbiol. 2007;65(2):413–24. 10.1111/j.1365-2958.2007.05794.x 17630972

[61] Kortmann J, Sczodrok S, Rinnenthal J, Schwalbe H, Narberhaus F. Translation on demand by a simple RNA-based thermosensor. Nucleic Acids Res. 2011;39(7):2855–68. 10.1093/nar/gkq1252 21131278 PMC3074152

[62] Nikolova EN, Al-Hashimi HM. Thermodynamics of RNA melting, one base pair at a time. RNA. 2010;16(9):1687–91. 10.1261/rna.2235010 20660079 PMC2924531

[63] Rinnenthal J, Klinkert B, Narberhaus F, Schwalbe H. Direct observation of the temperature-induced melting process of the Salmonella fourU RNA thermometer at base-pair resolution. Nucleic Acids Res. 2010;38(11):3834–47. 10.1093/nar/gkq124 20211842 PMC2887971

[64] Yakovchuk P, Protozanova E, Frank-Kamenetskii MD. Base-stacking and base-pairing contributions into thermal stability of the DNA double helix. Nucleic Acids Res. 2006;34(2):564–74. 10.1093/nar/gkj454 16449200 PMC1360284

[65] Dugar G, Svensson SL, Bischler T, Wäldchen S, Reinhardt R, Sauer M, et al The CsrA-FliW network controls polar localization of the dual-function flagellin mRNA in Campylobacter jejuni. Nat Commun. 2016;7:11667 10.1038/ncomms11667 27229370 PMC4894983

[66] Jervis AJ, Butler JA, Wren BW, Linton D. Chromosomal integration vectors allowing flexible expression of foreign genes in Campylobacter jejuni. BMC Microbiol. 2015;15:230 10.1186/s12866-015-0559-5 26497958 PMC4619491

[67] de Kok S, Stanton LH, Slaby T, Durot M, Holmes VF, Patel KG, et al Rapid and reliable DNA assembly via ligase cycling reaction. ACS Synth Biol. 2014;3(2):97–106. 10.1021/sb4001992 24932563

[68] Osman D, Waldron KJ, Denton H, Taylor CM, Grant AJ, Mastroeni P, et al Copper homeostasis in Salmonella is atypical and copper-CueP is a major periplasmic metal complex. J Biol Chem. 2010;285(33):25259–68. 10.1074/jbc.M110.145953 20534583 PMC2919089

[69] Corcoran CP, Cameron AD, Dorman CJ. H-NS silences gfp, the green fluorescent protein gene: gfpTCD is a genetically remastered gfp gene with reduced susceptibility to H-NS-mediated transcription silencing and with enhanced translation. J Bacteriol. 2010; 192(18):4790–3. 10.1128/JB.00531-10 20639321 PMC2937413

[70] Livak KJ, Schmittgen TD. Analysis of relative gene expression data using real-time quantitative PCR and the 2(-Delta Delta C(T)) Method. Methods. 2001;25(4):402–8. 10.1006/meth.2001.1262 11846609

