# Supplementary material for: RNA-based thermoregulation of a Campylobacter jejuni zinc resistance determinant
Source: PLoS Pathog. 2020 Oct 16;16(10):e1009008. doi: 10.1371/journal.ppat.1009008 (PMC7592916; doi:10.1371/journal.ppat.1009008)
Supplement: S1 Fig — The four active site residues for metal transport in YiiP [41] are highlighted in blue along with aligned conserved CzcD residues H73, D77, H179 and D183. Alignment was produced using Clustal Omega. (DOCX) [file ppat.1009008.s001.docx]

CzcDCj MYKFLSHEPLANKSCHHNHEEHSHEHHHSHADARSVDKKILKISLLMTFSMML--VQFIY 58

YiiPEc ---------------------------MNQSYGRLVSRAAIAATAMASLLLLIKIFAWWY 33

.:: .* *.: : : : :: ::: . : *

CzcDCj SILSNSLALLSDTL**H**MFS**D**VFALALSFLAIIAVEKWQDHQKTFGYFRLEVLVAFINALTI 118

YiiPEc T---GSVSILAALV**D**SLV**D**IGASLTNLLVVRYSLQPADDNHSFGHGKAESLAALAQSMFI 90

: .*:::*: :. : *: * .:*.: : *.:::**: : * *.*: ::: *

CzcDCj ILSALFIIYEAIEKFINPKEIDAKTMI-IVAILGFLVNGINALMMFKG---ANLENVNMK 174

YiiPEc SGSALFLFLTGIQHLISPTPMTDPGVGVIVTIVALICTII--LVSFQRWVVRRTQSQAVR 148

****:: .*:::*.*. : : **:*:.:: . * *: *: . :. ::

CzcDCj SAFL**H**MMS**D**LLGSLAVIIGGIVVYFSDIVYIDTILAIVLSILLLRWAIILLKQSANVLLE 234

YiiPEc ADML**H**YQS**D**VMMNGAILLALGLSWY-GWHRADALFALGIGIYILYSALRMGYEAVQSLLD 207

: :** **:: . *:::. : :: . *:::*: :.* :* *: : ::.: **:

CzcDCj SSPVDIEK--VRQVLLLNPSVDEVVDLHITQITNKMLVASMHLKVRV-CNLKEFEKLSQD 291

YiiPEc RALPDEERQEIIDIVTSWPGVSGAHDLRTRQSGPTRF-IQIHLEMEDSLPLVQAHMVADQ 266

: * *: : ::: *.*. . **: * . : .:**::. * : . ::::

CzcDCj LSHKLLHEFEIGHITI-------QPIRSENEI-- 316

YiiPEc VEQAILRRFPGSDVIIHQDPCSVVPREGKRSMLS 300

:.: :*:.* ..: * * ..:..:
