# Supplementary material for: RNA-based thermoregulation of a Campylobacter jejuni zinc resistance determinant
Source: PLoS Pathog. 2020 Oct 16;16(10):e1009008. doi: 10.1371/journal.ppat.1009008 (PMC7592916; doi:10.1371/journal.ppat.1009008)
Supplement: S2 Fig — (a) Clustal generated multiple sequence alignment of the Cj1164c/czcD intergenic region including Cj1164c stop codon and czcD start codon. The 38 sequence variants are numbered and presented in ranked order with the number of strains with that particular sequence indicated in brackets. The NCTC 11168 sequence belongs to the most common variant 1. (b) Predicted secondary structures for all 38 sequence variants of the Cj1164c/czcD intergenic region from 1626 C. jejuni strains using Mfold with standard parameters. (DOCX) [file ppat.1009008.s002.docx]

a

1 (717) TAATTTTT-ATAAAAATA--ACAAATATTTATATCCTT-TTTAGCTTAA-TATCTTAAAATGTCAAGCAAAAAAGGATATAAGATG

2 (250) TAATTTTT-ATAAAAATA--ACAAATATTTATATCCTT-TTTAGCTTAA-TATCTTAAAATGTCAAGCAAAAA-GGATATAAGATG

3 (125) TAATTTTT-ATAAGAATA--ACAAATATTTATATTCTT-TTTAGCTT-AATATCTTAAAATGTCAAGCAAAAA-GGATATAAAATG

4 (123) TAATTTTT-ATAAAAATA--ACAAATATTTATATCCTT-TTTAGCTTAA-TATCTTAAAATGTCAAGCAAAAAAGGATATAAAATG

5 (111) TAATTTT--ATAAAAATA--ACAAATATTTATATCCTT-TTTAGCTT-AATATCTTAAAATGTCAAGCAAAAA-GGATATAAGATG

6 (76) TAATTTTT-ATAAAAATA--ACAAAAATTTATATCCTT-TTTAACTTAA-TATCTTAAAATGTCAAGCAAAAA-GGATATAAGATG

7 (66) TAATTTTT-ATAAAAATA--ACAAATATTTATATCTTT-TTTAGCTTAA-TATCTTAAAATGTCAAGCAAAAA-GGATATAAGATG

8 (20) TAATTTTT-ATAAAAATA--ACAAATATTTATATTCTT-TTTAGCTT-AATATCTTAAAATGTCAAGCAAAAA-GGATATAAGATG

9 (17) TAATTTTT-ATAAAAATA--ACAAATATTTATATTCTT-TTTAGCTTAA-TATCTTAAAATGTCAAGCAAAAAAGGATATAAGATG

10 (16) TAATTTTT-ATAAAAATA--ACAAATATTTATATCCTT-TTTAGCTT-AATATCTTAAAATGTCAAGTAAAAA-GGATGTAAAATG

11 (15) TAATTTTT-ATAAGAATA--ACAAATATTTATATTCTT-TTTAACTT-AATATCTTAAAATGTCAAGCAAAAA-GGATATAAAATG

12 (12) TAATTTT--ATAAAAATA--ACAAATATTTATATCCTT-TTTAGCTTTAATATCTTAAAATGTCAAGCAAAAA-GGATATAAGATG

13 (11) TAATTTTTTATAAAAATA--ACAAATATTTATATCCTT-TTTAGCTT-AATATCTTAAAATGTCAAGTAAAAA-GGATGTAAAATG

14 (8) TAATTTTT-ATAAAAATA--ACAAATATTTATATCCTT-TTTAGCTTAA-TATCTTATAATGTCAAGCAAAAA-GGATATAAGATG

15 (6) TAATTTTT-ATAAAAATA--ACAAATATTTATATTCTT-TTTAGCTT-AATATCTTAAAATGTCAAGTAAAAA-GGATATAAAATG

16 (6) TAATTTTT-ATAAGAATA--ACAAATATTTATATTCTT-TTTAACTT-AATATCTTAAAATGTCAAGCAAAAA-GGATATAAAATG

17 (5) TAATTTTT-ATAAAAATA--ACAAATATTTATATCTTT-TTTAGCTT-AATATCTTAAAATGTCAAGTAAAAA-GGATATAAAATG

18 (5) TAATTTTTATTAAGAATA--ACAAATATTTATATTCTT-TTTAGCTT-AATATCTTAAAATGTCAAGCAAAAA-GGATATAAAATG

19 (4) TAATTTTTTATAAAAATA--ACAAATATTTATATCCTT-TTTAGCTT-AATATCTTAAAATGTCAAGCAAAAA-GGATATAAAATG

20 (4) TAATTTTT-ATAAAAATA--ACAAATATTTATATTCTT-TTTAGCTT-AATATCTTAAAATGTCAAGCAAAAA-GGATATAAAATG

21 (4) TAATTTTT-ATAAGAATA--ACAAATATTTATATTCTT-TTTAGCTT-AATATCTTAAAATGTCAAGCAAAAA-GGATATAAGATG

22 (3) TAATTTTT-ATAAAAATA--ACAAATATTTATATCCTT-TTTAGCTT-AATATCTTAAAATGTCAAGCAAAAA-GGATATAAAATG

23 (3) TAATTTT--ATAAAAATA--ACAAATATTTATATTCTT-TTTAGCTT-AATATCTTAAAATGTCAAGCAAAAA-GGATATAAGATG

24 (3) TAATTTTT-ATAAGAATA--ACAAATATTTATATTCTT-TTTAACTTAA-TATCTTAAAATGTCAAGCAAAAAAGGATATAAAATG

25 (2) TAATTTTT-ATAAAAATA--ACAAATATTTATATCCTT-TTTAGCTTAAATATCTTAAAATGTCAAGCAAAAA-GGATATAAGATG

26 (2) TAATTTTT-ATAAGAATA--ACAAATATTTATATTCTT-TTTAGCTT-AATATCTTAAAATGTCAAGTAAAAA-GGACATAAAATG

27 (1) TAATTTTT-ATAAAAATA--ACAAATATTTATATCCTT-TTTAGCTTAAATATCTTAAAATGTCAAGCAAAAAAGGATATAAGATG

28 (1) TAATTTTT-ATAAAAATA--ACAAATATTTATATCTTT-TTTAGCTTAA-TATCTTAAAATGTCAAGCAAAAAAGGATATAAGATG

29 (1) TAATTTTT-ATAAAAATA--ACAAATATTTATATCCTTGTTTAGCTTGA-TATCTTAAAATGTCAAGCAAAAAAGGATATAAGATG

30 (1) TAATTTTT-ATAAAAATA--ACAAATATTTATATCCTT-TTTAGCTTAA-TATGTTAAAATGTCAAGCAAAAA-GGATATAAGATG

31 (1) TAATTTTT-ATAAAAATA--ACAAATATTTATATCCTT-TTTAACTTAA-TATCTTAAAATGTCAAGCAAAAA-GGATATAAGATG

32 (1) TAATTTTT-ATAAAAATA--ACAAATATTTATATCCTT-TTTAGCTT-AATATCTTAAAATGTCAAGCAAAAA-GGATATAAAATG

33 (1) TAATTTTT-ATAAAAATA--ACAAATATTTATATCCTT-TTAAGCTTAA-TATCTTAAAATGTCAAGCAAAAA-GGATATAAGATG

34 (1) TAATTTT--ATAAAAATA--ACAAATATTTATATCCTT-TTTAGCTT-AATATATTAAAATGTCAAGCAAAAA-GGATATAAGATG

35 (1) TAATTTTT-ATAAAAATA---CAAATATTTATATTCTT-TTTAGCTT-AATATCTTAAAATGTCAAGCAAAAA-GGATATAAGATG

36 (1) TAATTTTT-ATAAAAATATAGCAAATATTTATATCCTT-TTTAGCTT-AATATCTTAAAATGTCAAGCAAAAA-GGATATAAAATG

37 (1) TAATTTTT-ATAAAAATA--ATAAATATTTATATTCTT-TTTAGCTT-AATATCTTAAAATGTCAAGTAAAAA-GGATATAAAATG

38 (1) TAATTTTT-ATAAGAATA--ACAAATATTTATATTCTA-TTTAGCTT-AATATCTTAAAATGTCAAGCAAAAA-GGATATAAAATG

******* *** **** *** ******** * ** * *** * *** *** ********* ***** *** *** ***

**b
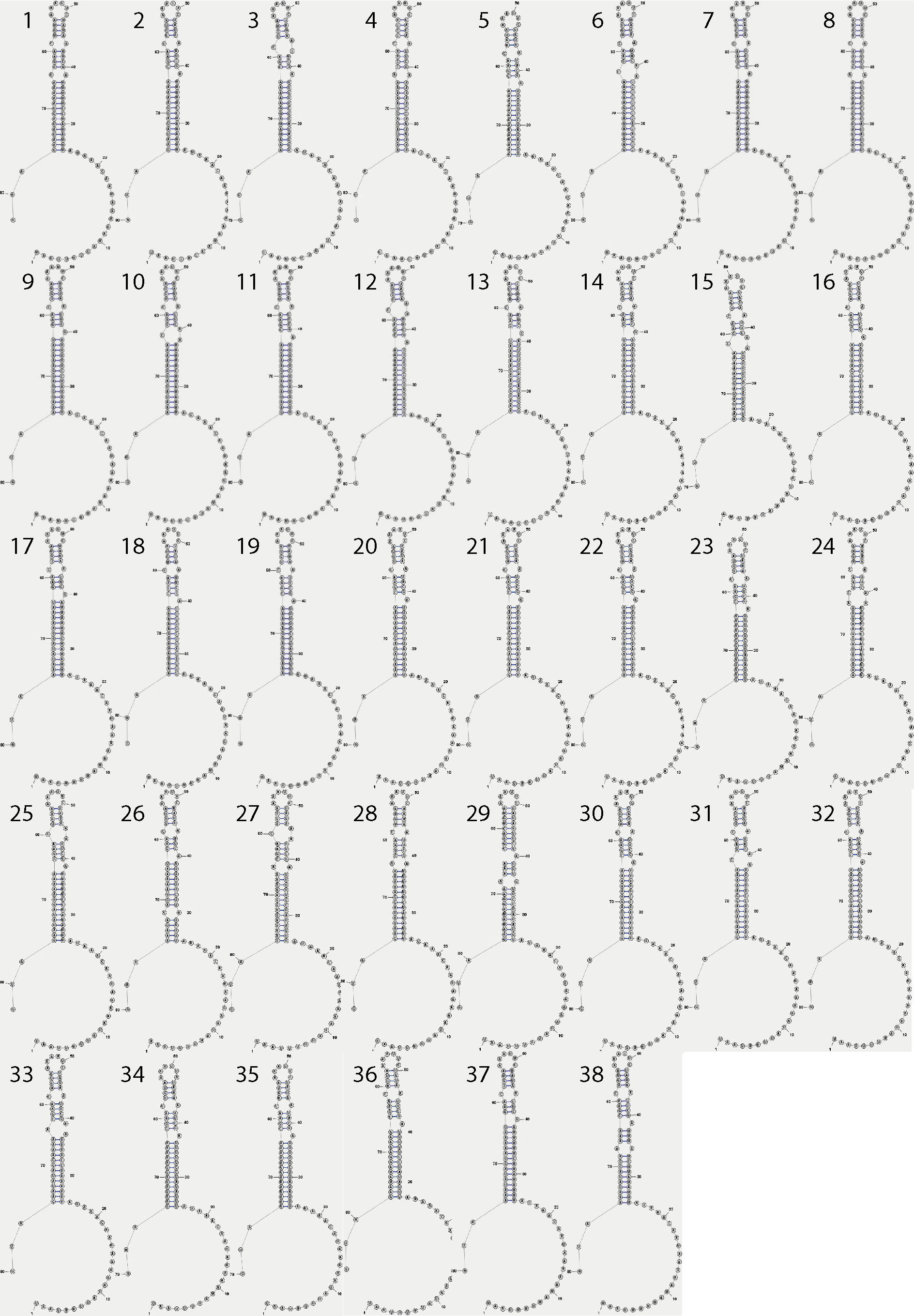
**
