# Supplementary figures and images for: RNA-based thermoregulation of a Campylobacter jejuni zinc resistance determinant
Source: PLoS Pathog. 2020 Oct 16;16(10):e1009008. doi: 10.1371/journal.ppat.1009008 (PMC7592916; doi:10.1371/journal.ppat.1009008)

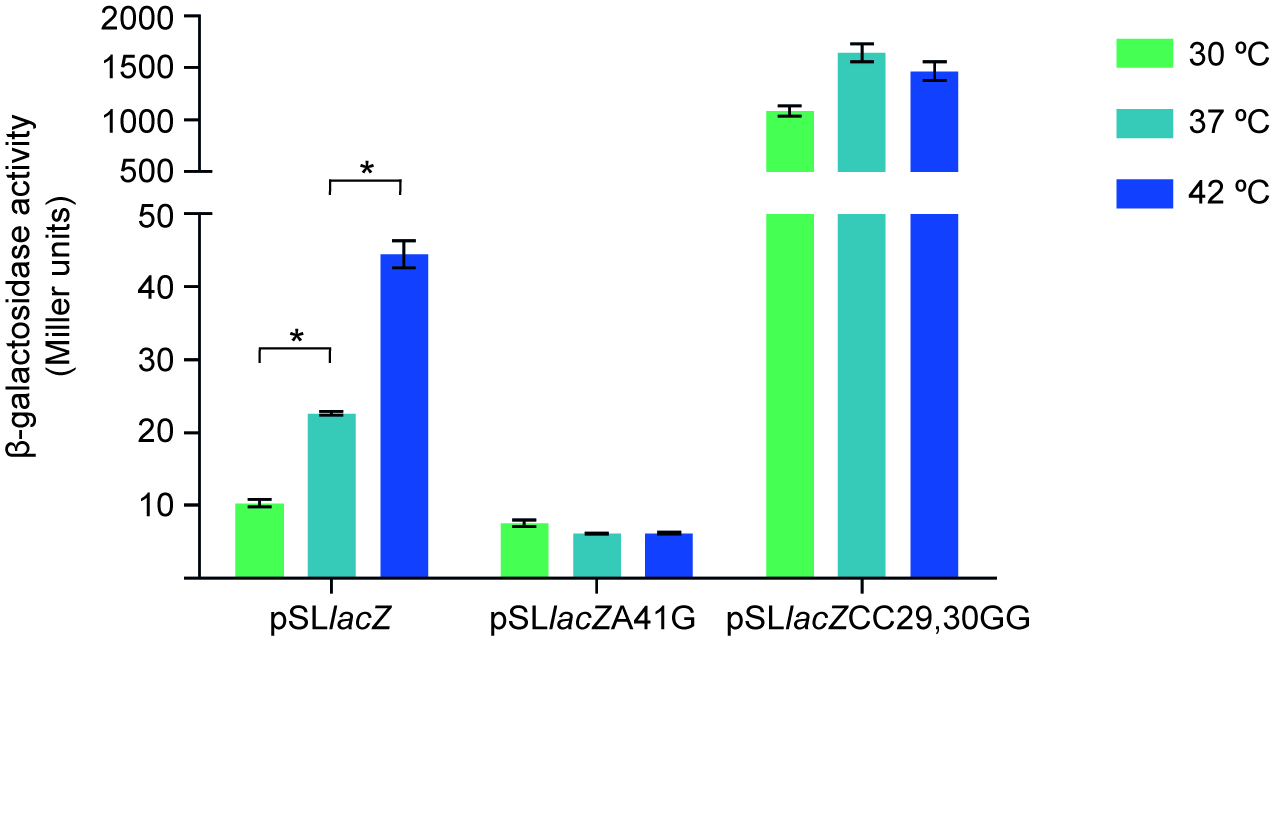

Supplement: S3 Fig — Strains of E. coli containing plasmids pSLlacZ, pSLlacZA41G or pSLlacZCC29,30GG as indicated were grown to mid log phase and expression of lacZ induced for two hours. Cells were harvested and β-galactosidase activities measured. Data presented are the mean of three (pSLlacZ), five (pSLlacZA41G) or two (pSLlacZCC29,30GG) biological repeats. Error bars represent standard error of the mean with * indicating a t-test p value of <0.05. (TIF) [file ppat.1009008.s003.tif]

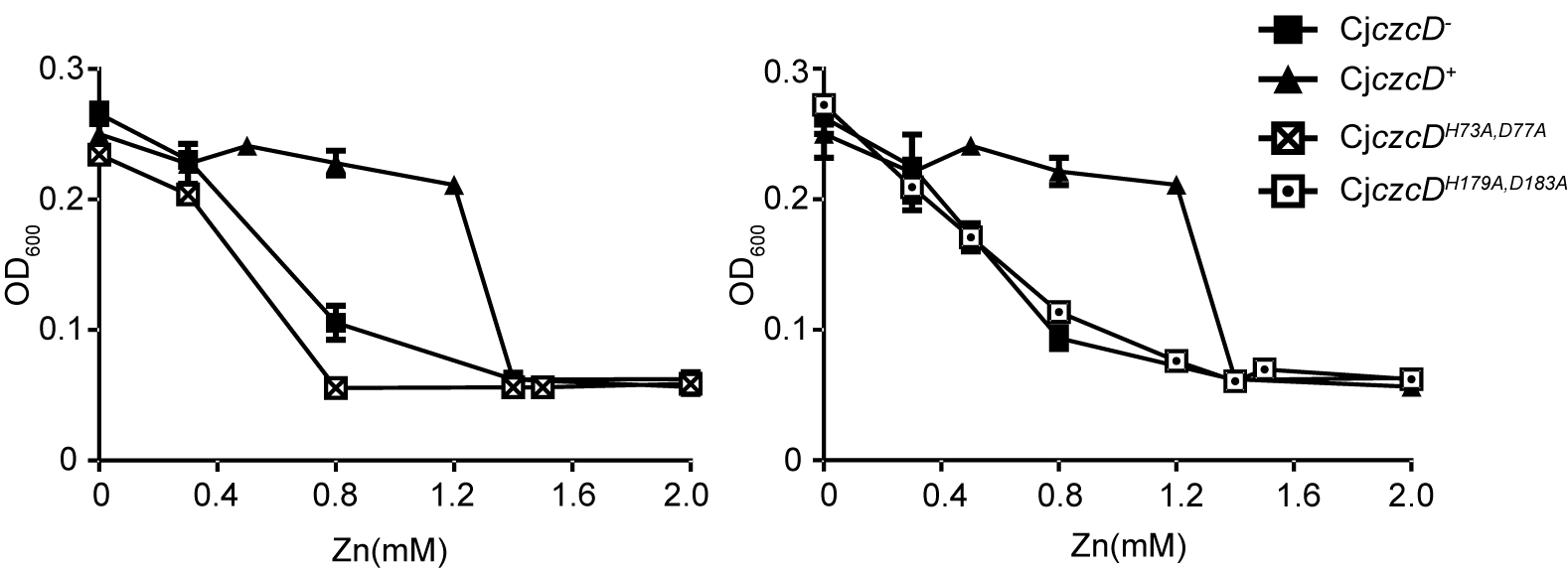

Supplement: S4 Fig — The CjczcD- strain was genetically complemented with wild-type czcD (CjczcD+) and with two mutant versions each with two alanine substitutions in the Zn(II) binding active site as identified in S1 Fig. Strains were grown in MEM-α containing varying Zn(II) concentrations (in 24 well tissue culture plates) from an initial OD600 of 0.05 at 37°C for 24 hours and the final OD600 recorded. Data points are the mean of three biological repeats, each with three technical repeats and the error bars indicate standard error of the mean of three biological repeats. (TIF) [file ppat.1009008.s004.tif]

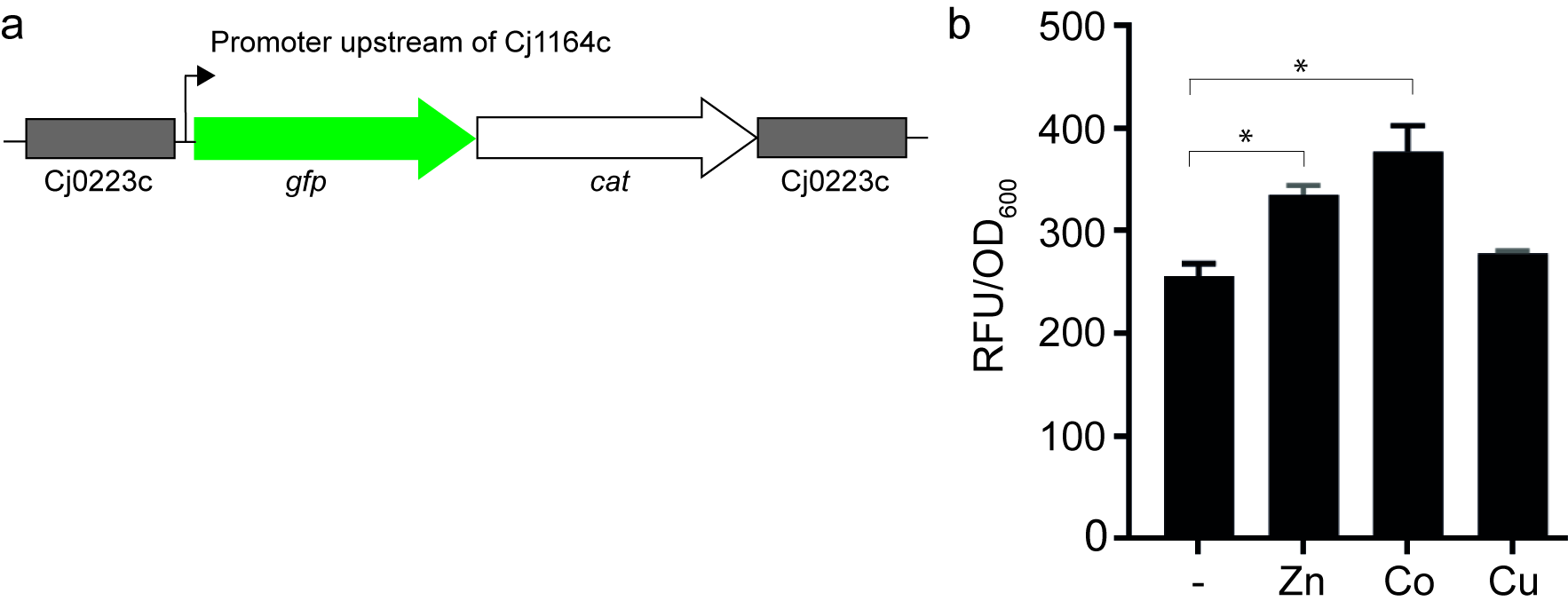

Supplement: S5 Fig — a. Diagrammatic representation of the region introduced onto the C. jejuni NCTC 11168 chromosome within the pseudogene Cj0223c to create strain CjPgfp. The arrowhead represents the σ70 promoter identified by RNAseq analyses [36,37] directing expression of Cj1164c to Cj1161c. The green arrow is the gfp reporter under control of the promoter and cat is a chloramphenicol resistance cassette. b. Cells of C. jejuni strain CjPgfp were grown for 24 hours in MEM-α media (-) or in MEM-α supplemented with sub-inhibitory concentrations of Zn(II) - 1.8 mM, Co (II) - 0.4 mM, or copper—0.4 mM as indicated. Relative fluorescence was calculated by dividing the fluorescence units by the optical density measured at 600 nm. Data presented are the average of three biological repeats in technical triplicate. Error bars represent the calculated standard error of the biological repeats and p values were calculated using the one-way ANOVA with * indicating p <0.005. (TIF) [file ppat.1009008.s005.tif]

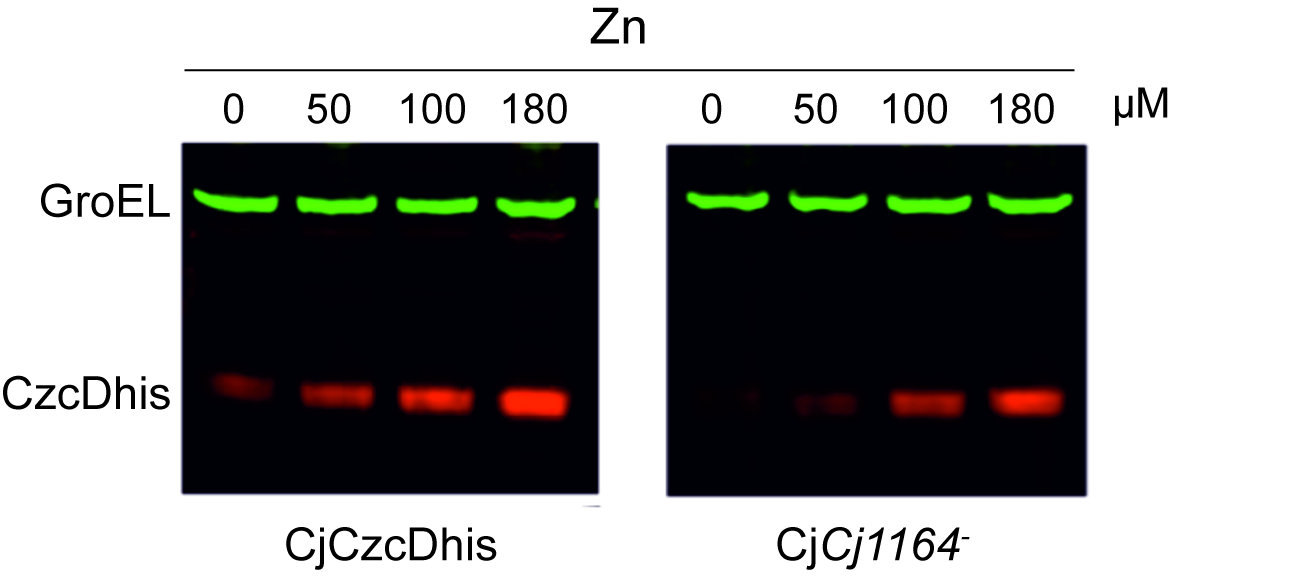

Supplement: S6 Fig — The CjCzcDhis strain and a derivative with a disrupted Cj1164c gene (CjCj1164-) were grown for 24 hours in MH broth with Zn(II) added at the indicated concentrations. Cells were harvested and following Western blotting of whole cell extracts, membranes were probed with anti-GroEL (green) as loading control and anti-histidine to detect CzcDhis (red). These data are representative of at least three replicate experiments. (TIF) [file ppat.1009008.s006.tif]
