# Supplementary material for: RNA-based thermoregulation of a Campylobacter jejuni zinc resistance determinant
Source: PLoS Pathog. 2020 Oct 16;16(10):e1009008. doi: 10.1371/journal.ppat.1009008 (PMC7592916; doi:10.1371/journal.ppat.1009008)
Supplement: S1 Table — (DOCX) [file ppat.1009008.s007.docx]

**Table S1. Minimum free energy values for the Cj1164c/*czcD* intergenic region transcript and site directed mutants characterized herein.**

| **Cj1164c/*czcD* intergenic region and mutant versions** | **Minimum free energy value or ΔG (kcal/mol)** |
| --- | --- |
| Wild type | -20.2 |
| CC29,30GG | -11.0 |
| CC29,30UU | -14.8 |
| A41G | -26.4 |
| A36U | -24.3 |
| G59C | -12.5 |
| G59C, C38G | -20.2 |
| U34A | -16.8 |
| U34A, A63U | -20.8 |

The minimum free energy values of the Cj1164c/*czcD* intergenic region and variants described above were obtained from the Mfold website using standard input parameters.
