# Supplementary material for: RNA-based thermoregulation of a Campylobacter jejuni zinc resistance determinant
Source: PLoS Pathog. 2020 Oct 16;16(10):e1009008. doi: 10.1371/journal.ppat.1009008 (PMC7592916; doi:10.1371/journal.ppat.1009008)
Supplement: S2 Table — (DOCX) [file ppat.1009008.s008.docx]

**Table S2. Oligonucleotides used in this study.**

| **Name** | **Sequence (5’ to 3’)** |
| --- | --- |
| Cj1163-UF | CGTTTGGCTTGATCGTGGAG |
| Cj1163-UR | CACTAAAGCTTCAAGACGAAAATACCCA |
| Cj1163-DF | GATTAAAGCTTTGATGATGTTTAAGGG |
| Cj1163-DR | TCCAAATTCATCTTCAAGAG |
| 0223upF | TTACACTTTGGAGGGGTAGC |
| endcatR | TTATTTATTCAGCAAGTCTTGTAATTC |
| 0223downf | GCAGGTTGATTATGCTTTGATCAAT |
| 0223downR | GGCACTCATTGTTACAGCTTC |
| Cj1163compF | ATTACAAGACTTGCTGAATAAATAAGTCAAGCAAAAAAGGATATAAGATG |
| Cj1163compR | ATTGATCAAAGCATAATCAACCTGCTTAAATTTCATTTTCACTCCTTATAGG |
| LCR_A F | CGCAGTGATGATTTGAAGGCTAA |
| LCR_A R | AGATATTAAGCTAAAAACCATATAAATATTTGTTAT |
| LCR_B F | TAAAATGTCAAGCAAAAAAGGATATAAGATG |
| LCR_B R | TTAGTGATGGTGATGGTGATGAATTTCATTTTCACTCCTTATAGG |
| LCR_C F | AAGGAGTGAAAATGAAATTTAAAGTAAAAAATG |
| LCR_C R | CTCAAAGCCATTTGGATATTTTGAGA |
| Bridge_BC | AGGAGTGAAAATGAAATTCATCACCATCACCATCACTAAAAGGAGTGAAAATGAAATTTAAAGTAAAAAATGTTAATTG |
| Bridge_AB | TTATAAAAATAACAAATATTTATATGGTTTTTAGCTTAATATCTTAAAATGTCAAGCAAAAAAGGATATAAGATGTATA |
| SL1 | GTGTGATAAAAATTTATACATCTTATATCCTTGAATTCATTTTTATAAAAATTAAAAGTCAAAAAGCTCGC |
| SL2 | GCGAGCTTTTTGACTTTTAATTTTTATAAAAATGAATTCAAGGATATAAGATGTATAAATTTTTATCACAC |
