# Supplementary material for: RNA-based thermoregulation of a Campylobacter jejuni zinc resistance determinant
Source: PLoS Pathog. 2020 Oct 16;16(10):e1009008. doi: 10.1371/journal.ppat.1009008 (PMC7592916; doi:10.1371/journal.ppat.1009008)
Supplement: S3 Table — (DOCX) [file ppat.1009008.s009.docx]

**Table S3. Strains used in this study.**

| **Strain** | **Relevant characteristics** | **Source** |
| --- | --- | --- |
| *E. coli* strains |  |  |
| DH5α | Cloning strain | Invitrogen |
| EcpMSLG | DH5α containing plasmid pMSLG | This study |
| EcΔSL | DH5α containing pMSLG*^ΔSL^* | This study |
| EcCC29,30GG | DH5α containing pMSLG*^CC29,30GG^* | This study |
| EcCC29,30UU | DH5α containing pMSLG*^CC29,30UU^* | This study |
| EcA36U | DH5α containing pMSLG*^A36U^* | This study |
| EcA41G | DH5α containing pMSLG*^A41G^* | This study |
| EcG59C | DH5α containing pMSLG*^G59C^* | This study |
| EcU34A | DH5α containing plasmid pMSLG*^U34A^* | This study |
| EcG59C,C38G | DH5α containing pMSLG*^G59C,C38G^* | This study |
| EcU34A,A63U | DH5α containing pMSLG*^U34A,A63U^* | This study |
| *C. jejuni* strains |  |  |
| NCTC 11168 | Wild-type | National Collection of Type Cultures, UK |
| CjCzcDhis | NCTC 11168 producing C terminal hexahistidine-tagged CzcD | This study |
| CjΔSL | CjCzcDhis strain with deletion within the stem loop region | This study |
| Cj^wt^ΔSL | NCTC 11168 with deletion within the stem loop region | This study |
| CjCC29,30GG | CjCzcDhis strain containing SDM version of stem loop region as indicated | This study |
| Cj*czcD^-^* | NCTC 11168 with inactivated *czcD* through insertion of a kanamycin resistance cassette *aphA* | This study |
| Cj*czcD^+^* | Cj*czcD^-^* strain genetically complemented through insertion of *czcD* within Cj0223c pseudogene and downstream of a chloramphenicol resistance cassette | This study |
| Cj*czcD^H73D77A^* | Cj*czcD^-^* strain genetically complemented through insertion of *czcD^H73D77^* within Cj0223c pseudogene and downstream of a chloramphenicol resistance cassette | This study |
| Cj*czcD^H179D183A^* | Cj*czcD^-^* strain genetically complemented through insertion of *czcD^H179D183^* within Cj0223c pseudogene and downstream of a chloramphenicol resistance cassette | This study |
| Cj*Cj1164^-^* | CjCzcDhis with inactivated *Cj1164c* through insertion of a promoter-less kanamycin resistance cassette *aphA* | This study |
| CjPgfp | NCTC 11168 with Cj1164c promoter, gfp and cat cassette introduced onto the chromosome within pseudogene Cj0223c | This study |
