# Supplementary material for: RNA-based thermoregulation of a Campylobacter jejuni zinc resistance determinant
Source: PLoS Pathog. 2020 Oct 16;16(10):e1009008. doi: 10.1371/journal.ppat.1009008 (PMC7592916; doi:10.1371/journal.ppat.1009008)
Supplement: S4 Table — (DOCX) [file ppat.1009008.s010.docx]

**Table S4. Plasmids used in this study.**

| **Plasmid** | **Description** | **Source** |
| --- | --- | --- |
| pMAL-c5X |  | New England Biolabs |
| pMG | *gfp*^TCD^ cloned into NdeI/SalI digested pMAL-c5X | This study |
| pMSLG | *Cj1164*c-*czcD* intergenic region (reverse complement of nucleotides 1095025 to 1095084 in AL111168) cloned between *malE* and *gfp* of pMG using oligonucleotides SL1 and SL2 | This study |
| pMSLG*^C29,309GG^* | pMSLG with SDM of stem loop bases as indicated | This study |
| pMSLG*^A36U^* | pMSLG with SDM of stem loop bases as indicated | This study |
| pMSLG*^A41G^* | pMSLG with SDM of stem loop bases as indicated | This study |
| pMSLG*^ΔSL^* | pMSLG with bases 1-24 inclusive of stem loop region removed | This study |
| pMSLG*^G59C^* | pMSLG with SDM of stem loop bases as indicated | This study |
| pMSLG*^U34A^* | pMSLG with SDM of stem loop bases as indicated | This study |
| pMSLG*^G59C,C38G^* | pMSLG with SDM of stem loop bases as indicated | This study |
| pMSLG*^U34A,A62U^* | pMSLG with SDM of stem loop bases as indicated | This study |
| pBAD*lacZ* | pMLBAD derivative containing *lacZ* directionally cloned into NheI and XbaI sites | This study |
| pSL*lacZ* | pBAD*lacZ* derivative containing SL*czcD* cloned immediately upstream of *lacZ* | This study |
| pSL*lacZ*^A41G^ | pSL*lacZ* derivative with SL*czcD* SDM as indicated | This study |
| pSL*lacZ*^CC29,30GG^ | pSL*lacZ* derivative with SL*czcD* SDM as indicated | This study |
| pCJC1P*gfp* | pCJC1 [66] derivative containing the Cj1164 associated promoter and *gfp* immediately upstream of the chloramphenicol resistance cassette | This study |
